# A tetrahedral molecular cage with a responsive vertex†
†Electronic supplementary information (ESI) available. See DOI: 10.1039/c9sc02047k


**DOI:** 10.1039/c9sc02047k

**Published:** 2019-06-13

**Authors:** Christopher C. Pattillo, Jeffrey S. Moore

**Affiliations:** a Department of Chemistry , University of Illinois at Urbana-Champaign , Urbana , Illinois 61801 , USA . Email: jsmoore@illinois.edu

## Abstract

Dynamic covalent chemistry (DCC) is a widely used method for the self-assembly of three-dimensional molecular architectures. The orthogonality of dynamic reactions is emerging as a versatile strategy for controlling product distributions in DCC, yet the application of this approach to the synthesis of 3D organic molecular cages is limited. We report the first system which employs the orthogonality of alkyne metathesis and dynamic imine exchange to prepare a molecular cage with a reversibly removable vertex. This study demonstrates the rational and controlled application of chemical orthogonality in DCC to prepare organic cages of expanded functionality which respond to chemical stimuli.

## Abstract 2

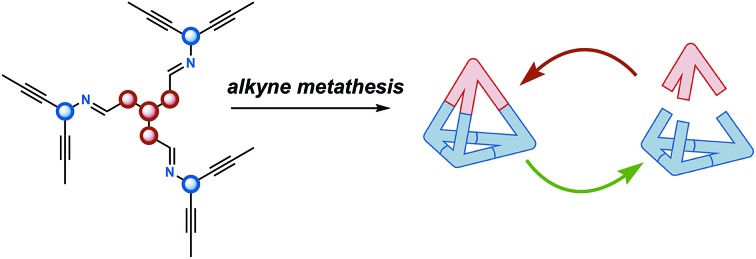
The first system to combine the orthogonality of alkyne metathesis and dynamic imine exchange is reported for the preparation of a molecular cage with a reversibly removable vertex.

## Introduction

**Fig. 1:**
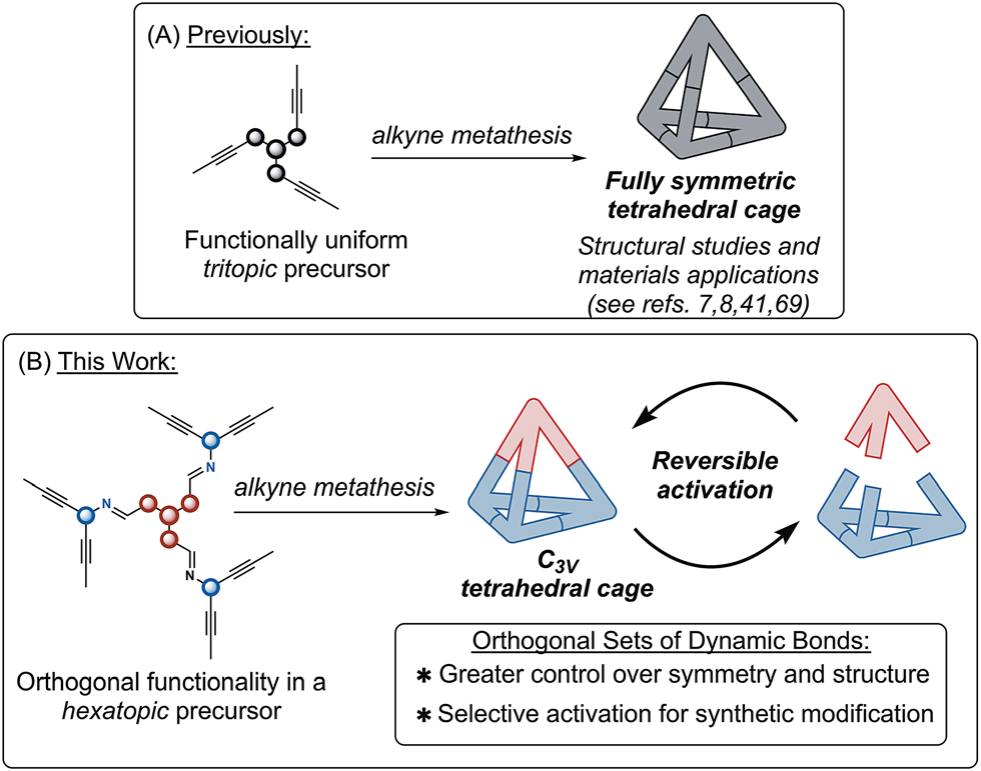
A molecular cage is prepared *via* orthogonal dynamic covalent reactions. The inclusion of orthogonal dynamic bonds allows this cage to respond to chemical stimuli in a controlled manner.

Recent improvements in alkyne metathesis (AM) have led to expanded applications in both total synthesis and materials chemistry.^1^–^6^ Alkyne metathesis is one among a number of reversible reactions utilized in dynamic covalent chemistry (DCC), and is particularly useful for the synthesis of 3D organic molecular cages.^2^,^7^–^10^ DCC employs reversible covalent bonds to self-assemble complex structures from simpler building blocks.^10^ The DCC toolbox comprises a number of orthogonal reactions,^11^ including alkyne/olefin metathesis,^2^,^3^ and imine/hydrazone,^12^–^18^ boronic ester^19^–^21^ and disulfide/dithioacetal exchange.^22^–^28^ Orthogonality in DCC is frequently leveraged in dynamic combinatorial library synthesis, where modification of reaction conditions (*e.g.* pH) can alter product distributions through selective activation of dynamic bonds.^25^,^26^,^29^ The ability to ‘turn-on’ a subset of dynamic functionalities affords a synthetic handle to reversibly shift product distributions and generate modified structures through post-synthetic transformations.^30^–^32^ The utility of orthogonal DCC has also been applied to the synthesis of organic cages and macrocycles through both simultaneous and sequential activation of orthogonal bonds to generate complex architectures from simple precursors.^33^–^38^ Recent success by our lab and others in the synthesis of organic cages *via* alkyne metathesis has led us to pursue both synthetic modifications and materials applications of these shape-persistent structures (Fig. 1A).^7^–^9^,^39^–^41^ After surveying the literature, we were motivated by the apparent lack of examples combining alkyne metathesis with orthogonal dynamic chemistries in multitopic DCC.^42^,^43^ The development of highly active and functional group tolerant AM catalysts further demonstrates that this methodology is poised to expand into new chemical space.^1^,^6^,^44^–^51^ We have therefore chosen to pursue synthetic strategies which leverage orthogonal dynamic chemistries to prepare novel molecular cages. Herein, we demonstrate the preparation of a molecular cage incorporating two orthogonal dynamic bonds, each of which are selectively activated (Fig. 1B).

Inspired by the established utility of dynamic imine chemistry,^12^–^16^,^52^–^56^ we chose to investigate the feasibility of combining this orthogonal bond with alkyne metathesis. Two key considerations stood out at the onset of this study; namely, whether imine functionality is *chemically* compatible with AM and *geometrically* compatible with the alkyne bond to afford a reasonable 3D cage structure. Pathway complexity in multitopic DCC means that seemingly small variations in precursor structure can lead to large changes in product distribution.^8^,^57^ As such, substituting the trigonal imine bond for one or more linear alkyne bonds could alter the precursor structure such that no stable product configuration is available. Drawing inspiration from Zhang *et al.* who demonstrated the synthesis of macrocycles and cages through tandem imine exchange and olefin metathesis,^36^,^37^ we hypothesized that a sufficiently preorganized alkyne metathesis precursor could be generated through coupling of alkyne subunits *via* imine condensation.

## Results and discussion

**Scheme 1:**
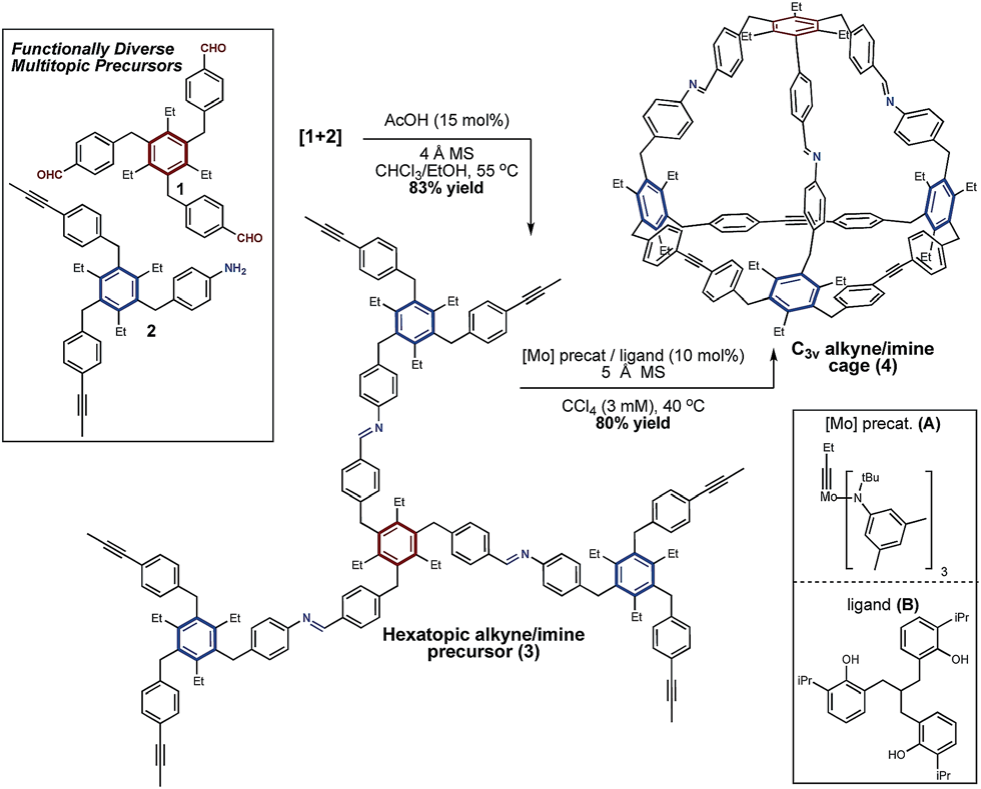
Synthesis of a *C*_3v_ symmetric tetrahedral organic cage *via* a tandem imine condensation/alkyne metathesis strategy.

To begin, we prepared functional precursors **1** and **2** to demonstrate the compatibility of AM with the imine functional group (Scheme 1). These precursors are analogous to those which afford tetrahedral cages *via* alkyne metathesis, where the 1,3,5-arrangement of a hexasubstituted arene aids in preorganizing the structure towards cage formation.^7^,^58^–^60^ We next coupled **1** and **2***via* imine condensation to afford a single *hexatopic* alkyne metathesis precursor **3** which we hypothesized is sufficiently preorganized to undergo cyclization to a cage (Scheme 1). The coupling of **1** and **2** proceeds in good yield and precursor **3** isolated by precipitation from methanol. This precursor also exhibits good solubility in organic solvents (*i.e.* CHCl_3_ and CCl_4_) which are well-suited for alkyne metathesis reactions.

Having prepared imine-linked precursor **3** we investigated its reactivity under alkyne metathesis conditions. Given the lack of examples of imine functionality in AM,^42^ a key consideration was the selection of an appropriate metathesis catalyst. Zhang and co-workers have demonstrated that tridentate, phenol-based supporting ligands generate highly active and functionally tolerant molybdenum metathesis catalysts.^44^,^45^,^51^ We began our investigations using ligand **B** from which the active catalyst is generated *in situ* upon stirring with molybdenum precatalyst **A**.^44^,^61^ In our preliminary experiments we were delighted to find that upon exposure of precursor **3** to this catalyst system at mild temperature (40 °C) a product putatively assigned to the imine-linked cage **4** was present as determined by Gel Permeation Chromatography (GPC) and MALDI-MS analysis. Upon further optimization, we found that using 10 mol% molybdenum catalyst **A**/ligand **B** in CCl_4_ (3 mM) at 40 °C afforded mixed imine/alkyne cage **4** in 80% yield and approximately 96% purity (Scheme 1). As is standard in alkyne metathesis, 5 Å molecular sieves were added to remove 2-butyne and drive the reaction forward.^62^ The metathesis reaction proceeds well at useful scales (*e.g.* 100 mg of precursor) and the product is easily isolated by precipitation and filtration. GPC analysis supports conversion of **3** to **4**, with higher-molecular weight species (*ca.* 4%) also present in the isolated material (see Fig. S2†). MALDI-MS analysis of the isolated product suggests **4** and the presence of a dimeric structure, which may be a catenated cage-structure or a dimer of precursor **3** (Fig. S1†). Despite numerous attempts to obtain an X-ray crystal structure of **4**, including several poorly diffracting single crystals, we were unable to obtain a data set suitable for structure refinement. Cage **4** was further characterized by ^1^H and ^13^C NMR (see ESI†). ^1^H NMR analysis of isolated **4** shows the loss of the propynyl signal in precursor **3** and a downfield shift indicating successful metathesis. Two characteristic doublets for the alkyne-linked ‘base’ of **4** are nearly identical in chemical shift to the tetrahedral cage we have reported previously^7^ (*δ*7.45/*δ*7.02 ppm and *δ*7.44/*δ*6.99 ppm respectively), and the presence of four additional doublets are consistent with the differentiated imine-linked vertex. ^1^H–^1^H COSY analysis allowed for assignment of the aromatic proton signals (see ESI†). ^13^C NMR analysis also shows the loss of the propynyl signal of **3**. Cage **4** was further characterized by a number of 2D NMR experiments (*i.e.* HSQC, HMBC, and NOESY, see ESI†).

**Fig. 2:**
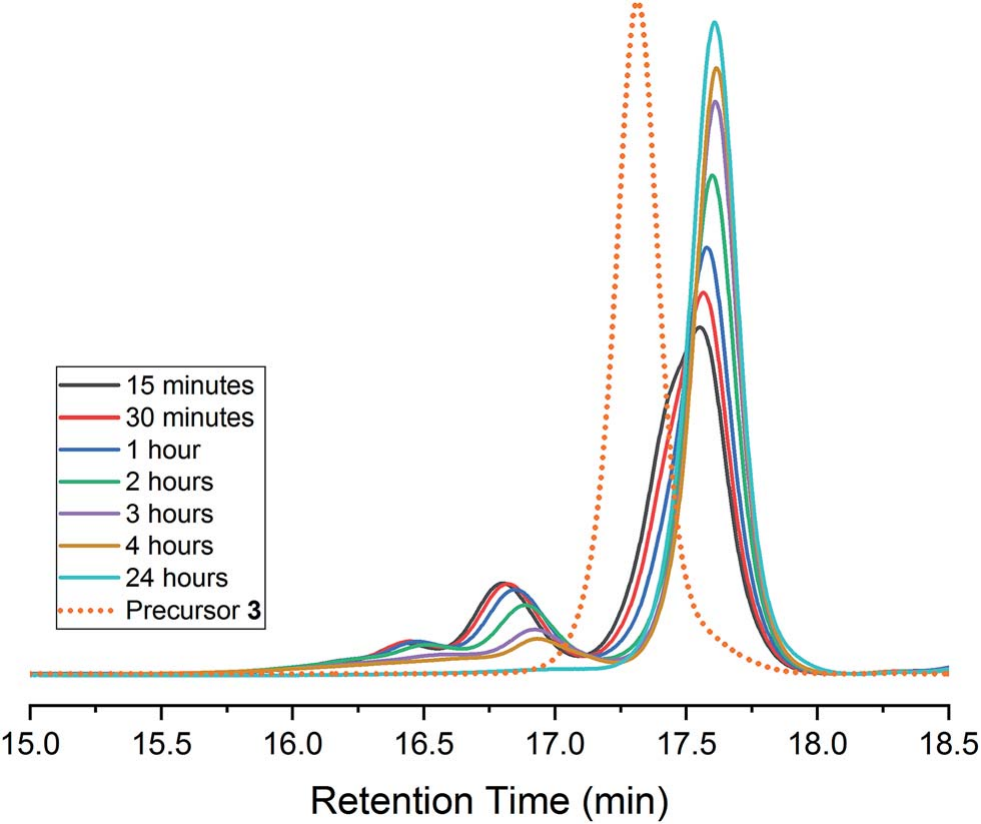
GPC analysis of the reaction of **3** to **4**. Note the formation of higher molecular weight products which ‘self-correct’ over time. Traces were normalized by area.

A common characteristic of DCC reactions is the ability for these systems to ‘self-correct’ to a discrete product.^8^,^16^,^53^,^63^–^65^ In many systems employing alkyne metathesis, high molecular weight oligomeric/polymeric products are often formed at early reaction times and gradually convert to a single product.^8^,^39^ In previous studies on the synthesis of *T*_d_ symmetric cages from tritopic precursors, we observed the formation of oligomeric intermediates which proceed on a pathway to a discrete cage.^8^ Given that **4** results from a single hexatopic precursor, we wondered if its formation bypasses any oligomeric intermediates and only proceeds *via* sequential intramolecular metathesis steps.^66^–^68^ We monitored the progress of the AM reaction of **3** to **4** by GPC and observed that, even at the dilute 3 mM reaction concentration, oligomeric products are formed which reversibly correct to a discrete product (Fig. 2). In addition to higher molecular weight species (16–17 min retention time), early time points display a ‘shouldering’ which may be intramolecular metathesis products as precursor **3** converts to cage. The dimeric product observed in the isolated **4** may account for the peak observed at *ca.* 17 minutes retention time. The relative proportion of higher molecular weight products also appears to be lower than we have observed in the metathesis reaction of other tritopic precursors.^8^ This may suggest that the predominant pathway for conversion of **3** to **4** consists of intramolecular metathesis steps. Thus, even when alternate reaction pathways are available for **3**, the reversible nature of AM allows for these intermediates to return to a pathway that leads to **4**.^7^,^8^ These results also suggest that a tetrahedral cage is the favoured reaction outcome for hexasubstituted tritopic and hexatopic precursors (*i.e.*, **1**, **2**, and **3**) which are geometrically preorganized in a manner that biases the reaction pathway to favour tetrahedral cage formation.^7^

With cage **4** in hand, we turned our attention to identifying conditions for selective activation of the single all-imine connected vertex. In the context of cage synthesis, methods which allow for controlled modification of cage geometry, functionality, and cavity size offer the potential to tailor these properties for specific host–guest interactions.^40^,^56^,^69^–^71^ We therefore hypothesized that the presence of orthogonal subsets of dynamic bonds in **4** would prove useful for its selective modification. We were particularly inspired by the efficiency of Sc(iii) catalysed transimination reactions.^16^,^53^,^72^ In a seminal publication, Lehn and co-workers demonstrated that transimination is effectively catalysed by Sc(OTf)_3_ in the presence of alkyl/aryl amines.^72^ The resulting equilibrium distribution has a tendency to favour the imine formed from the most basic amine.^72^ Though transimination reactions do proceed in the absence of a Lewis or Brønsted acid catalysts,^73^–^75^ Lehn has reported rate enhancements of up to five orders of magnitude in the presence of Sc(OTf)_3_.^72^ The high efficiency of this Lewis acid-catalysed transimination was thus attractive as a method to activate the imine-linked vertex of cage **4**.

**Fig. 3:**
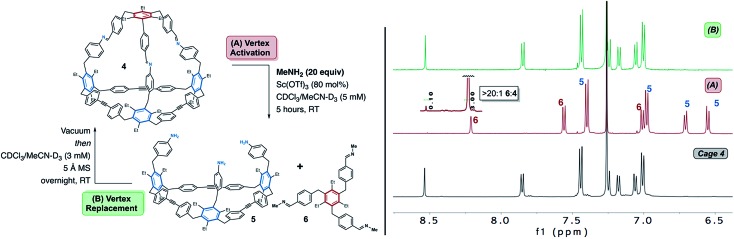
The imine-linked vertex of cage **4** is activated through Sc(iii) or Brønsted acid catalysed transimination. Left: Exposure of cage **4** to Sc(OTf)_3_ in the presence of methylamine results in formation of macrocycle **5** and tris-imine **6**. Right: Comparison of NMR spectra of starting cage (bottom), disassembled cage reaction mixture (middle), and re-assembled cage reaction under Sc(iii) catalysis (top). The reassembly reaction mixture was passed through a short plug of basic alumina and concentrated to dryness. Peak labels correspond to the disassembly products (macrocycle **5** and tris-imine **6**). NMR analysis performed in CDCl_3_, 500 MHz, 25 °C.

We began our investigations under the hypothesis that a high concentration of a basic alkyl amine would result in an equilibrium that favours removal of the imine vertex of **4**. Methylamine, which is commercially available as a THF solution, was selected for cage disassembly studies. Methylamine is basic and highly volatile which allows for simple removal *via* high-vacuum (see below). Cage **4** was subjected to 20 equiv. of methylamine in CDCl_3_/MeCN-D_3_ with Sc(OTf)_3_ as catalyst (Fig. 3). After 5 h at RT, ^1^H NMR analysis of the reaction mixture was consistent with disassembly of cage **4** to macrocycle **5** and tris-imine **6** (Fig. 3). NMR analysis shows an upfield shift of the imine proton signal of cage **4** from *δ*8.52 ppm to *δ*8.21 ppm, consistent with transimination from an aryl- to an alkyl-substituted imine (Fig. 3 and S5†).^72^ Compound **6** was independently synthesized to confirm its assignment (Fig. S4†). MALDI-MS analysis of the reaction mixture confirms the presence of macrocycle **5** (Fig. S6†). While the NMR spectra suggest that disassembly is nearly complete, detectable levels of the residual cage **4** were also observed in the MALDI spectrum (Fig. S6†). MALDI also suggests detectable levels of partially disassembled cage **4**, in which one and two imines, respectively, have reacted with methylamine (Fig. S6†). From the NMR spectra, we conclude the disassembly affords a >20 : 1 ratio of **6** : **4** (see Fig. 3 inset and S5†). Upon addition of Sc(OTf)_3_ to the reaction mixture, some cloudiness was observed indicating that formation of insoluble oligomeric products may be a competing side-reaction during the disassembly process. Brønsted acid catalysis also effectively disassembles **4**. Subjecting the cage to similar conditions employing catalytic trifluoroacetic acid afforded comparable results to the reaction performed using Sc(OTf)_3_ as catalyst (see Fig. S7†). Other alkyl amines (*i.e.*, *n*-propylamine) are also capable of disassembling cage **4** under both Sc(OTf)_3_ and TFA catalysis (see Fig. S7† for results with *n*-propylamine disassembly using TFA).

Having identified conditions which disassemble **4** we next sought to determine whether the cage could be subsequently reassembled after its conversion to **5** and **6**. This would demonstrate that a viable assembly pathway is available from two starting points; *i.e.* dynamic alkyne metathesis and dynamic imine exchange reactions. In early reassembly experiments using *n*-propylamine, we were initially intrigued to observe partial reassembly of cage **4** after removal of excess amine and stirring the reaction mixture overnight. Given that dynamic imine exchange is in equilibrium, we recognized that even after removal of excess amine, an equilibrium would still exist between cage **4** and compounds **5** and **6**.^72^ We hypothesized that this equilibrium would shift through *in situ* removal of methylamine remaining after transimination of **5** and **6** to cage **4**. In dynamic alkyne metathesis, 5 Å molecular sieves (MS) are used to sequester residual 2-butyne and drive the reaction forward.^62^ We hypothesized that 5 Å MS would also effectively sequester residual methylamine. In order to probe the feasibility of reassembly, the cage disassembly reaction was performed as described above, using volatile methylamine under scandium catalysis (Fig. 3). After stirring for five hours, the resulting reaction mixture was dried under high vacuum for one hour to remove excess methylamine. After re-dissolving the residue from the disassembly process and allowing the reaction to stir overnight at room temperature in the presence of 5 Å molecular sieves, ^1^H NMR analysis of the reaction was consistent with reassembly of cage **4** (Fig. 3). MALDI-MS and ^1^H NMR analysis of the remaining material shows that the cage is reformed efficiently, with no additional products observed by MALDI-MS (Fig. S6†).

The results of this study demonstrate that assembly of **4** is achieved through dynamic alkyne metathesis from a hexatopic precursor **3** and through dynamic imine exchange from **5** and **6**. The fact that **4** is accessed from both starting points through orthogonal DCC reactions demonstrates that it possesses a structural stability that is favoured and kinetically viable for both processes. As indicated by the results in Fig. 2, the formation of alternate reaction products does not preclude the formation of cage **4** from either starting point; instead, these intermediates are capable of returning to a pathway to a discrete cage structure. We imagine that the ability to assemble discrete molecular cages *via* orthogonal dynamic bonds will open new opportunities not only for synthesis of diverse molecular cages, but also for controlled modification of these architectures.

## Conclusion

In this report we have demonstrated the synthesis of a *C*_3v_ symmetric organic molecular cage assembled from two dynamic covalent reactions. By combining orthogonal alkyne and imine bonds the resulting cage possesses an imine-linked vertex which is removed and replaced through Sc(iii) catalysed transimination. To the best of our knowledge this is the first example of orthogonal dynamic covalent chemistry being combined with alkyne metathesis for the synthesis of organic molecular cages and the first example of imine functionality being tolerated in an AM reaction. This work not only demonstrates a strategy for preparing cages of altered symmetry but opens new avenues for the preparation of discrete cages which are modified selectively. We believe that this study complements existing strategies for molecular cage synthesis and will allow for the preparation of architectures with greater control over their symmetry and functionality. Further studies into the preparation of complex nanostructures using this method, as well as their applications in selective host–guest chemistry, are ongoing.

## Conflicts of interest

There are no conflicts to declare.

## Supplementary Material

Supplementary information
