# Supplementary material for: A tetrahedral molecular cage with a responsive vertex
Source: Chem Sci. 2019 Jun 13;10(29):7043–8. doi: 10.1039/c9sc02047k (PMC6676470; doi:10.1039/c9sc02047k)
Supplement: Supplementary file 1 [file SC-010-C9SC02047K-s001.pdf]

Electronic Supplementary Material (ESI) for Chemical Science.
This journal is © The Royal Society of Chemistry 2019

Supporting Information

A Tetrahedral Molecular Cage with a Responsive Vertex

Christopher C. Pattillo and Jeffrey S. Moore*

Department of Chemistry, University of Illinois Urbana-Champaign, Champaign, IL 61820

*Corresponding author (jsmoore@illinois.edu)

S.1 General Considerations	S2
S.2 Synthesis and Characterization of Compounds	S2-S8
S.3 Time Course Experiments	S9
S.3 Cage Disassembly/Reassembly Experiments	S10-S14
S.4 ^{1}H and ^{13}C NMR Spectra	S15-S43
S.5 References	S44

S.1 General Considerations

All reactions were performed in oven (c.a. 165 °C) or flame-dried glassware under an atmosphere of dry argon or nitrogen unless otherwise noted. All solvents used were either anhydrous commercial grade (Aldrich/Fisher) or purified by a solvent purification system unless otherwise noted. Reaction chloroform was filtered through a pad of basic alumina and dried over 3 Å molecular sieves prior to use. All alkyne metathesis reactions were conducted in an argon-filled glovebox in oven-dried glassware, using anhydrous (Aldrich), argon-degassed solvents. All reagents were purchased from commercial sources and used without further purification. Molybdenum(VI) propylidyne precatalyst **A** and triphenol ligand **B** were prepared according to published literature procedures.[1,2] Molecular sieves (5 Å powdered) were dried in a vacuum oven at 200 °C for 5 days prior to use in alkyne metathesis reactions.[3] Chromatographic purifications were conducted via MPLC on a Biotage Isolera 1 using Silicycle SiliaSep cartridges (230-400 mesh, 40-63 µm). Column separation conditions are reported in column volumes (CV) of gradient solvent mixtures. Analytical gel permeation chromatography (GPC) was performed in THF on a Tosoh Ecosec HLC8320GPC at 40°C equipped with a reference column (6.0 mm ID x 15 cm), guard column (6.0 mm ID x 4.0 cm x 5 µm) and two analytical columns (7.8 mm ID x 30 cm x 5 µm). The reference flowrate was set to 0.5 mL/min and the analytical column flowrate set to 1.0 mL/min. ^{1}H and ^{13}C nuclear magnetic resonance spectra (NMR, 500 MHz) were recorded at room temperature (298 K) and chemical shifts were referenced to the residual solvent peak. Matrix-assisted laser desorption/ionization (MALDI) mass spectrometry was performed on a Bruker Daltonics UltrafleXtreme MALDI using DCTB matrix. MALDI spectra and GPC traces were plotted using OriginPro 2018 software. GPC traces were normalized by area and baseline corrected. ^{1}H and ^{13}C NMR were processed using MestReNova software v12.0.4-22023. Reported yields are of isolated material which in some cases were corrected for trace residual solvent.

S.2 Synthesis and Characterization of Compounds

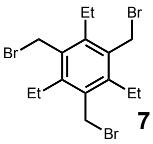

7

1,3,5-tribromobenzyl-2,4,6-triethylbenzene (7): In a 500 mL round bottom flask equipped with stirbar, $ZnBr_2$ (41.08 g, 182.4 mmol, 3 equiv) and paraformaldehyde (16.4 g, 546.1 mmol, 9 equiv) were combined and dissolved in AcOH (100 mL). 1,3,5-triethylbenzene (11.5 mL, 60.8 mmol, 1 equiv) was added, followed by HBr (33% w/w in AcOH, 100 mL). The flask was sealed with a glass stopper and the joint was wrapped with electrical tape. The mixture was then heated to 100 °C overnight behind a blast-shield. The reaction mixture was briefly cooled and poured over ice to afford a brown precipitate. After diluting the mixture with water and stirring briefly, the brown precipitate was vacuum filtered and washed thoroughly with methanol (ca. 300 mL) and dried under vacuum to afford **7** as a white powder (20 g, 45.3 mmol, 75% yield).

^{1}H NMR (500 MHz, CDCl$_3$): δ 4.58 (s, 6H), 2.95 (q, *J* = 7.6 Hz, 6H), 1.35 (t, *J* = 7.7 Hz, 9H).

^{13}C NMR (126 MHz, CDCl$_3$): δ 145.15, 132.81, 28.70, 22.90, 15.76.

HRMS (EI+): $C_{15}H_{21}Br_3$ [M+] calcd 437.9193, found 437.9190.

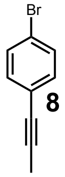

1-bromo-4-(prop-1-yn-1-yl)benzene (8): In a flame-dried 500 mL round bottom flask equipped with stirbar, 1-bromo-4-iodobenzene (15 g, 53 mmol, 1 equiv), $Pd(PPh_3)_2Cl_2$ (1.86 g, 2.65 mmol, 5 mol%) and CuI (1 g, 5.3 mmol, 10 mol%) were combined and the reaction flask was backfilled three times with argon. Toluene (130 mL) and Et_3N (130 mL) were then added and the mixture was stirred briefly. The reaction mixture was then cooled to -78 ^{o}C using a dry ice/acetone bath and propyne gas was added to the reaction mixture. (**Note:** excess propyne was used. The propyne gas was bubbled through the mixture and into the headspace of the reaction from a small lecture bottle via an 18-gauge metal needle with an argon balloon as pressure release. The gas was added in approximately 4 ten-second bursts.) The reaction mixture was allowed to stir at -78 ^{o}C for 5 minutes after which the bath was removed, and the mixture was stirred at RT for 24 hours. The dark black reaction mixture was then filtered through a short plug of silica gel with Et_2O and DCM and concentrated directly onto silica gel. The reaction was purified *via* silica gel flash chromatography with a gradient of 2 CV hexane to 2 CV 1% EtOAc/Hexane (CV = column volume) to afford **2** as a clear yellow oil (8.38 g, 42.9 mmol, 81% yield).

^{1}H NMR (500 MHz, CDCl$_3$): δ 7.41 (d, *J* = 8.4 Hz, 2H), 7.24 (d, *J* = 8.4 Hz, 2H), 2.03 (s, 3H).

^{13}C NMR (126 MHz, CDCl$_3$): δ 133.09, 131.54, 123.14, 121.72, 87.26, 78.91, 4.48.

HRMS (EI+): C_9H_7Br [M+] calcd 193.9731, found 193.9737.

4,4'-((5-(bromomethyl)-2,4,6-triethyl-1,3-phenylene)bis(methylene))bis(prop-1-yn-1-ylbenzene) (9): In a 100 mL round bottom flask with stirbar, magnesium turnings (881 mg, 36.24 mmol, 4 equiv) were flame-dried under vacuum. The flask was backfilled with argon and a small amount of I_2 in THF (36 mL) was added and stirred at RT for 5 min, after which **8** (3.5 g, 18.12 mmol, 2 equiv) was added portion-wise. The resulting green/brown solution was stirred for 15 minutes at RT then at 70°C for two hours. In a separate flame-dried 200 mL round-bottom flask, **7** (4 g, 9.06 mmol, 1 equiv) and $CuBr(SMe)_2$ (372.5 mg, 1.8 mmol, 0.2 equiv) were combined and the flask was purged with N_2 for 15 minutes. THF (54 mL) was then added, and the Grignard solution of **8** was added via cannula while stirring at 70 ^{o}C and the resulting mixture was stirred overnight at 70 ^{o}C. The reaction was quenched slowly with 50 mL of 1M HCl and diluted with water. The layers

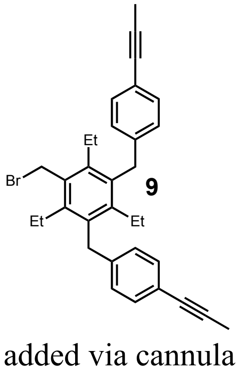

were then separated, and the aqueous layer was extracted 1x with DCM. The combined organic layers were dried with Na_2SO_4, filtered through silica and then concentrated onto silica. The reaction mixture was purified *via* silica gel flash column chromatography using a gradient of 1 CV 7% DCM/hexanes, 2 CV 10% DCM/hexanes, 2 CV 15% DCM/hexanes, 2 CV 20% DCM/hexanes, 2 CV 30% DCM/hexanes to afford compound **9** as a white solid (639.06 mg, 1.25 mmol, 14% yield). ***Note:*** *compound **9** coelutes with an unknown impurity which appears to be an isomer. The isolated material can be carried forward and further purified after conversion to **2**.*

^{1}H NMR (500 MHz, $CDCl_3$): δ 7.26 (d, J = 8.4 Hz, 4H), 6.88 (d, J = 8.0 Hz, 4H), 4.66 (s, 2H), 4.08 (s, 4H), 2.71 (q, J = 7.3 Hz, 4H), 2.37 (q, J = 7.5 Hz, 2H), 2.03 (s, 6H), 1.18 (t, J = 7.6 Hz, 6H), 0.97 (t, J = 7.5 Hz, 3H).

^{13}C NMR (126 MHz, $CDCl_3$): δ 144.11, 142.23, 140.52, 134.30, 131.69, 127.80, 121.64, 85.41, 79.75, 34.53, 30.54, 23.93, 23.28, 15.60, 14.97, 4.47.

HRMS (EI+): $C_{33}H_{35}Br$ [M+] calcd 510.1922, found 510.1930.

4-(2,4,6-triethyl-3,5-bis(4-(prop-1-yn-1-yl)benzyl)benzyl)aniline (2): In an oven-dried 40 mL reaction vial equipped with a septum-cap and a stirbar, magnesium turnings (126 mg, 5.2 mmol, 4 equiv) were dried under vacuum with a heat gun. After cooling to room temperature and backfilling with argon, THF (6.5 mL) was added followed by 50 µL of 1,1-dibromoethane and the mixture was stirred for five minutes. 4-Bromo-*N,N*-bis(trimethylsilyl)aniline (0.75 mL, 2.65 mmol, 2 equiv, Aldrich) was then added in one portion and the mixture was stirred briefly at room temperature then at 70 °C for 1 hour. In a separate oven-dried reaction vial, **9** (665 mg, 1.3 mmol, 1 equiv) and CuI (25 mg, 0.115 mmol, 0.1 equiv) were combined and backfilled twice with argon. THF (8.5 mL) was then added, followed by the Grignard solution and the mixture was stirred overnight at 70 °C. The reaction was quenched with 1M HCl (10 mL) and stirred vigorously for one hour. After diluting with 50 mL of 1M NaOH and extracting 3x with EtOAc, the combined organic layers were washed with brine, dried with Na_2SO_4 and filtered through a short silica plug. The mixture was then concentrated onto silica gel and purified via silica gel flash chromatography using a gradient of 10%-15%-20%-25%-30%-40%-50%-100% Et_2O/pentane. The purified product was isolated as an off-white foam (485.3 mg, 0.92 mmol, 71% yield).

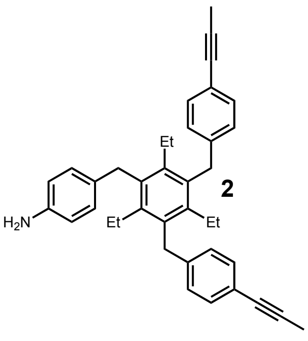

2

^{1}H NMR (500 MHz, $CDCl_3$): δ 7.26 (d, J = 8.1 Hz, 4H), 6.90 (d, J = 8.0 Hz, 4H), 6.76 (d, J = 8.1 Hz, 2H), 6.59 (d, J = 8.3 Hz, 2H), 4.10 (s, 4H), 4.01 (s, 2H), 3.53 (s, 2H), 2.41 (dq, J = 21.6, 7.5 Hz, 6H), 2.03 (s, 6H), 1.03 (dt, J = 12.5, 7.4 Hz, 9H).

^{13}C NMR (126 MHz, $CDCl_3$): δ144.27, 141.51, 141.23, 141.12, 134.72, 133.65, 131.58, 131.32, 128.63, 127.82, 121.39, 115.43, 85.24, 79.84, 34.63, 33.84, 23.78, 15.31, 15.26, 4.48.

HRMS (ESI) m/z: $C_{39}H_{42}N$ $[M+H]^+$ calculated 524.3317, found 524.3301.

4,4',4''-((2,4,6-triethylbenzene-1,3,5-triyl)tris(methylene))tribenzaldehyde (1): In a round bottom flask, magnesium turnings (659 mg, 27.12 mmol, 12 equiv) were flame dried under vacuum and the flask was backfilled with argon. THF (45 mL), dibromoethane (50 μL) and 4-bromobenzaldehyde dimethyl acetal (2.3 mL, 13.6 mmol, 6 equiv) were sequentially added and stirred at 70 °C for 2 hours. In a separate flame-dried round bottom flask, **7** was added and the flask was backfilled x2 with argon. THF (23 mL) and Li_2CuCl_4 (0.1 M/THF, 4.5 mL, 0.45 mmol, 0.2 equiv) were added. The Grignard solution was added, and the reaction was stirred overnight at 70 °C. The reaction was then slowly quenched with 1 M HCl at RT (ca. 50 mL) and stirred for one hour. The aqueous layer was separated and the organic layer extracted x3 with Et_2O, then dried with $Na_2S_2O_4$, filtered and concentrated onto silica gel. The crude reaction was purified using silica gel flash chromatography using an acetone/hexanes gradient. Compound **5** was isolated as a white solid (625.18 mg, 1.21 mmol, 54% yield).

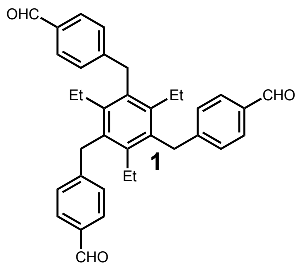

^{1}H NMR (500 MHz, $CDCl_3$): δ 9.97 (s, 3H), 7.79 (d, *J* = 8.2 Hz, 6H), 7.17 (d, *J* = 7.9 Hz, 6H), 4.23 (s, 6H), 2.42 (q, *J* = 7.5 Hz, 6H), 1.05 (t, *J* = 7.5 Hz, 9H).

^{13}C NMR (126 MHz, $CDCl_3$): δ 191.93, 148.87, 141.82, 134.80, 133.50, 130.14, 128.45, 35.17, 23.98, 15.18.

HRMS (ESI) m/z: $C_{36}H_{37}O_3$ $[M+H]^+$ 517.2743; Found 517.2720.

(1*E*,1'*E*,1''*E*)-1,1',1''-(((2,4,6-triethylbenzene-1,3,5-triyl)tris(methylene))tris(benzene-4,1-diyl))tris(*N*-methylmethanimine) (6): In a 1-dram vial, aldehyde **1** (25 mg, 43 μmol, 1 equiv) was dissolved in $CHCl_3$ and a small drop of glacial acetic acid was added. To the solution was then added methylamine (2M in THF, 0.65 mL, 1.29 mmol, 30 equiv) and the reaction was stirred overnight at room temperature. The mixture was then filtered through a small plug of neutral alumina, and dried with Na_2SO_4. After filtration, the mixture was concentrated to dryness and dried under vacuum to afford **6** as an off-white foam (31.6 mg, quantitative yield).

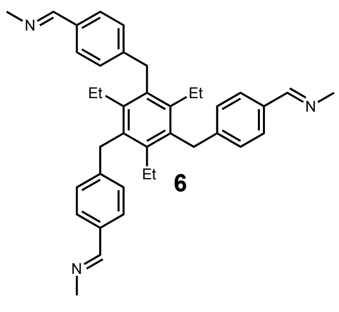

^{1}H NMR (500 MHz, CDCl$_3$) δ 8.23 (d, *J* = 1.8 Hz, 3H), 7.58 (d, *J* = 8.0 Hz, 6H), 7.03 (d, *J* = 8.0 Hz, 6H), 4.16 (s, 6H), 3.49 (d, *J* = 1.6 Hz, 9H), 2.42 (q, *J* = 7.5 Hz, 6H), 1.06 (t, *J* = 7.4 Hz, 9H).

^{13}C NMR (126 MHz, CDCl$_3$): δ 162.39, 144.29, 141.55, 134.20, 133.82, 128.11, 48.33, 34.73, 23.83, 15.26.

HRMS (ESI): Compound **6** appears to be unstable and decompose during ESI analysis. Aldehyde **1** was detected (517.2717, $[M+H]^+$) as well as the mono-imine ($C_{37}H_{40}NO_2$ $[M+H]^+$ calcd 530.3059, found 530.3024) and a mass of 544.2839 which may be the bis-imine isomer ($C_{38}H_{43}N_2O$ $[M+H]^+$ calcd 543.3375).

1*E*,1'*E*,1''*E*)-1,1',1''-(((2,4,6-triethylbenzene-1,3,5-triyl)tris(methylene))tris(benzene-4,1-diyl))tris(*N*-(4-(2,4,6-triethyl-3,5-bis(4-(prop-1-yn-1-yl)benzyl)benzyl)phenyl)methanimine)

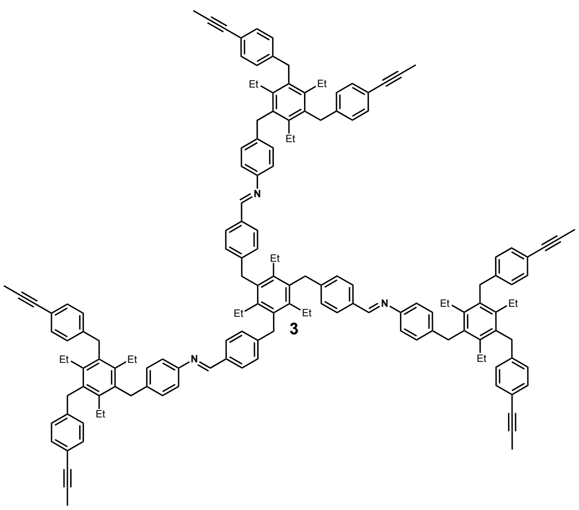

(3): In a 20 mL reaction vial equipped with a stir bar and a septum-topped cap, compound **2** (517.47 mg, 0.98 mmol, 4.35 equiv) and **1** (0.22 mmol, 117.3 mg, 1 equiv) were combined along with 4 Å MS (200 mg) and dissolved in $CHCl_3$ (1.1 mL) and EtOH (1.1 mL). Acetic acid (2.0 μL, 34.1 μmol, 15 mol%) was added and the mixture was stirred at 55 °C overnight. The mixture was diluted with chloroform and filtered through a 0.45 μm PTFE syringe filter into a 40 mL vial. MeOH (ca. 35 mL) was then added which resulted in the formation of a white precipitate. The mixture was vortexed thoroughly and the solid was collected via vacuum filtration through a 0.45 μm nylon membrane filter. The solid was washed thoroughly with methanol, followed by a 30% $CHCl_3$/MeOH solution and finally methanol. The solid was dried under vacuum to afford an off-white solid which was re-dissolved in minimal $CHCl_3$. Ethanol (ca. 35 mL) was added to re-precipitate. The solid was again collected via vacuum filtration and washed thoroughly with ethanol (ca. 50 mL), followed by a 30% $CHCl_3$/EtOH solution and finally ethanol. The material was dried under vacuum to afford **6** as an off-white powder (382.3 mg, 0.188 mmol, 83% yield). We recommend storing **3** long term in a -20 °C freezer over Drierite. *Note: NMR/MALDI-analysis indicate the presence of a bis-imine isomer and trace residual* ***2****. The purity of* ***3*** *was determined to be 96% by ^{1}H NMR.*

^{1}H NMR (500 MHz, CDCl$_3$): δ 8.41 (s, 3H), 7.79 (d, *J* = 8.0 Hz, 6H), 7.26 (d, *J* = 8.1 Hz, 12H), 7.15 – 7.05 (m, 12H), 6.98 (d, *J* = 8.2 Hz, 6H), 6.90 (d, *J* = 8.0 Hz, 12H), 4.21 (s, 6H), 4.12 (s,

6H), 4.10 (s, 12H), 2.43 (tt, $J = 15.5, 7.6$ Hz, 24H), 1.99 (s, 18H), 1.08 (t, $J = 7.4$ Hz, 9H), 1.06 – 0.97 (m, 27H).

^{13}C NMR (126 MHz, $CDCl_3$): δ 159.59, 150.05, 145.17, 141.65, 141.52, 141.40, 141.17, 139.17, 134.43, 134.23, 133.79, 131.62, 129.03, 128.56, 128.26, 127.81, 121.46, 121.05, 85.29, 79.82, 34.94, 34.67, 34.35, 23.92, 23.85, 23.82, 15.27, 15.26, 4.45.

MS-MALDI-TOF (DCTB matrix) m/z: $C_{153}H_{154}N_3$ [M+H$^+$] calcd 2033.21, found 2033.16.

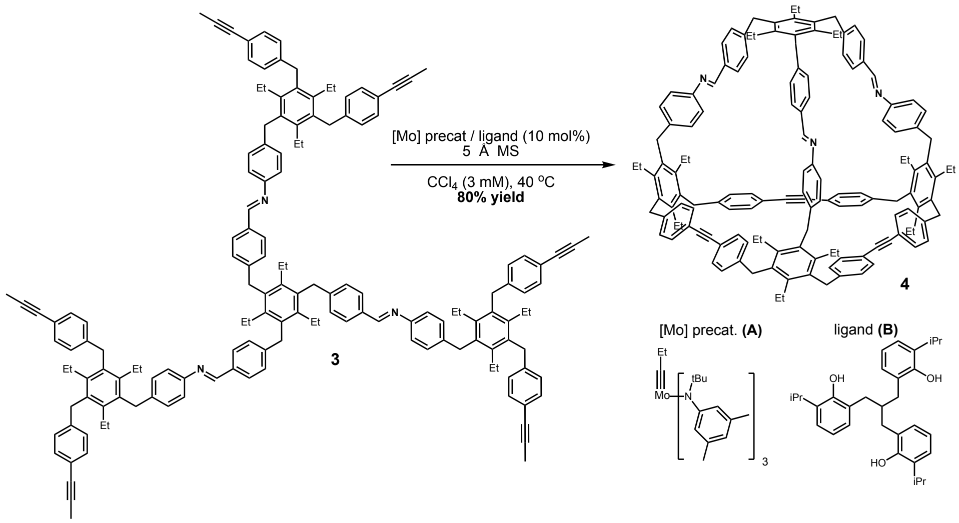

General Procedure for Synthesis of Imine/Alkyne Cage (4): In an argon-filled glovebox, precursor **3** (100 mg, 49 μmol, 1 equiv) was weighed into an oven-dried 40 mL reaction vial equipped with a septum cap. 5 Å MS (300 mg, 1g/mmol of propynyl functionality) were then added, followed by 14 mL of CCl_4. In a separate oven-dried 2-dram vial, molybdenum precatalyst **A** (3.3 mg, 4.9 μmol, 10 mol%) and triphenol ligand **B** (2.3 mg, 4.9 μmol, 10 mol%) were combined in 2 mL CCl_4. Both solutions were stirred for 30 minutes at room temperature. To the solution of precursor **3** was then added the catalyst solution via Pasteur pipette, and the reaction mixture (3 mM total in **3**) was capped and stirred at 40 °C in an aluminum block overnight (ca. 23 hours). The reaction was removed from the glovebox and filtered through a small plug of neutral alumina with excess $CHCl_3$. The mixture was concentrated *in-vacuo* and the resulting solid was dissolved in $CHCl_3$ and filtered through a small plug of Celite into a 40 mL vial. The solution was then concentrated and the residue dissolved in minimal (ca. 2 mL) $CHCl_3$. Excess ethanol (ca. 35 mL) was then added to afford a fine white precipitate. The solid was collected via filtration through a 0.45 μm nylon membrane filter and washed with ethanol and a solution of 30% $CHCl_3$/EtOH and finally methanol. The resulting off-white solid was dried under high-vacuum to afford cage **4** as a beige solid (80% average yield, 96% purity by GPC, average of two runs).
Run 1: 75.9 mg, 0.0406 mmol, 83% yield; **Run 2:** 69.5 mg, 0.0372 mmol, 76% yield.

^{1}H NMR (500 MHz, Chloroform-*d*): δ 8.54 (s, 3H), 7.86 (d, *J* = 7.9 Hz, 6H), 7.45 (d, *J* = 8.0 Hz, 12H), 7.25 (d, *J* = 6.4 Hz, 6H), 7.19 (d, *J* = 7.9 Hz, 6H), 7.07 (d, *J* = 8.1 Hz, 6H), 7.02 (d, *J* = 8.0 Hz, 12H), 4.20 (s, 6H), 4.14 (s, 18H), 2.57 – 2.34 (m, 24H), 1.24 – 1.15 (m, 36H).

^{13}C NMR (126 MHz, $CDCl_3$): δ 158.22, 149.05, 144.60, 141.67, 141.59, 141.45, 141.26, 139.09, 134.70, 134.04, 133.59, 133.45, 131.88, 129.13, 128.37, 128.10, 127.66, 121.61, 121.10, 89.31, 35.17, 34.82, 34.55, 23.93, 23.81, 15.13, 15.10, 14.99.

MS-MALDI-TOF (DCTB Matrix) m/z: $C_{141}H_{136}N_3$ $[M+H]^+$ calcd 1871.07, found 1871.99.

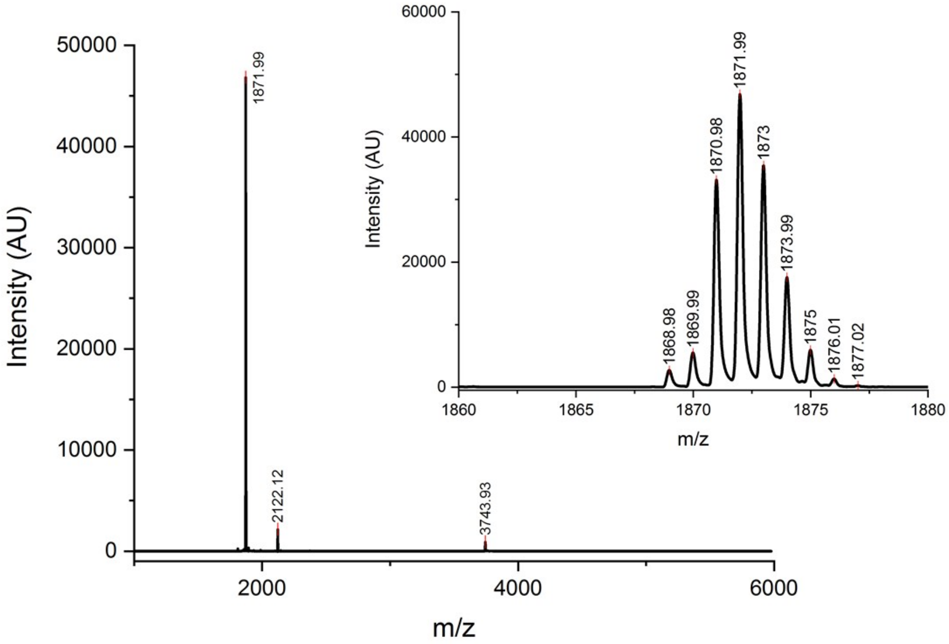

Figure S1. MALDI-TOF spectrum of isolated cage **4** (positive ion mode, DCTB matrix). Note the observed mass of a dimeric product (3743.93). We propose that the observed mass at m/z 2122.12 is an adduct of cage **4** with the DCTB matrix (see ref. 5).

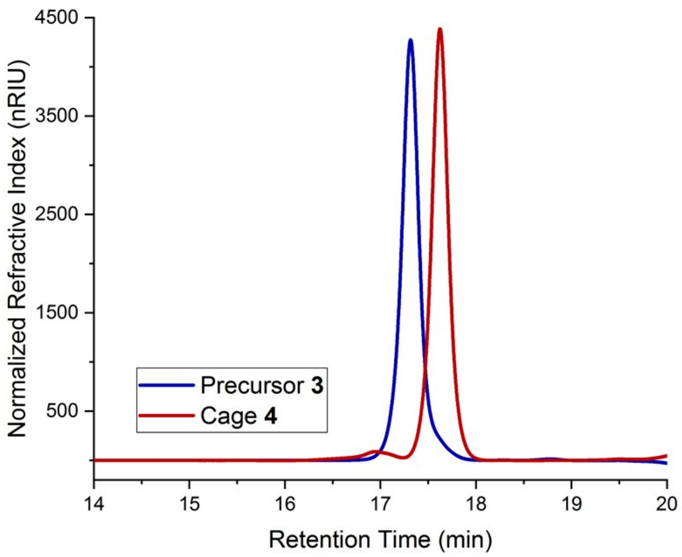

Figure S2. GPC traces of precursor **3** and isolated cage **4**. Percent purity of **4** was estimated by dividing the peak integration of higher molecular weight species (~17 min) by the total peak integration from retention time of 16- 18 minutes. Traces were normalized by area.

S3. Reaction Time-Course Experiments

Following the general procedure for synthesis of cage **4**, precursor **3** (50 mg, 24.5 µmol, 1 equiv) was reacted with [Mo] catalyst **A** (1.6 mg, 2.45 µmol, 10 mol%) and ligand **B** (2.45 µmol, 1.1 mg, 10 mol%) in 8.2 mL CCl_4 (3 mM) at 40°C in an argon-filled glovebox. Aliquots (200 µL) were removed at the appropriate timepoints and placed in sealed, air-filled ½ dram vials to quench. The aliquots were dried under high vacuum to remove CCl_4 and diluted with 1 mL of THF. After filtration through a 0.45 µm syringe filter, the samples were analyzed by GPC to characterize the product distribution.

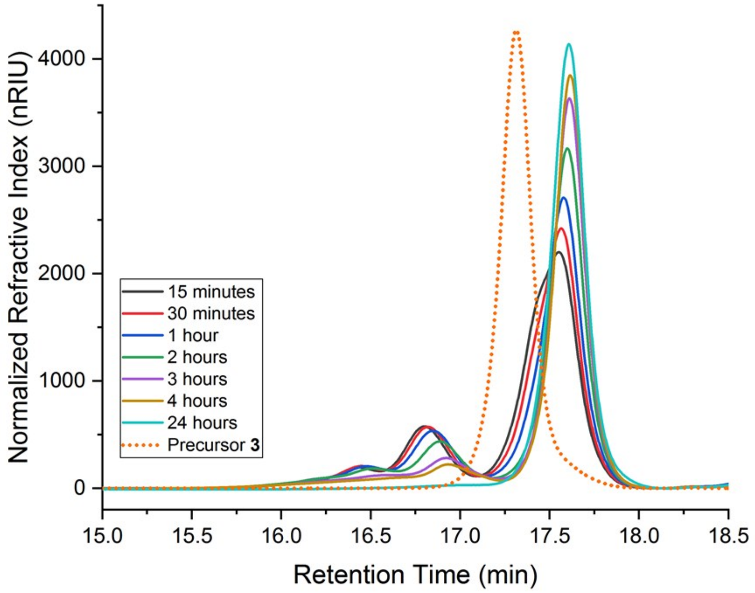

Figure S3. GPC time course of the AM reaction of precursor **3**. Note the formation of higher molecular weight products which are consumed over the course of the reaction. Traces normalized by area.

S4. Cage Disassembly and Reassembly Experiments

A. Cage Disassembly/Reassembly Using Scandium Triflate

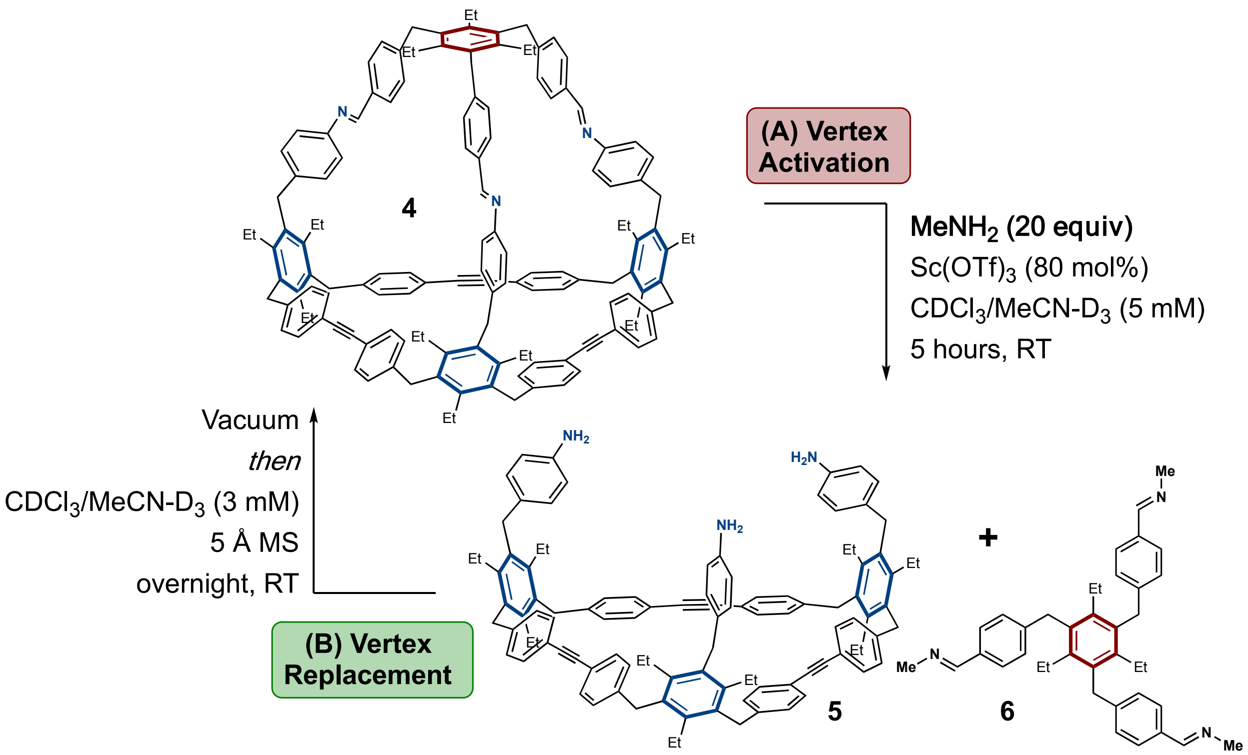

Cage Disassembly: In a 1-dram reaction vial, cage **4** (29.7 mg, 15.8 µmol, 1 equiv) was dissolved in $CDCl_3$ (3 mL, 5 mM) and stirred for five minutes to dissolve. In an argon-filled glovebox, $Sc(OTf)_3$ (6.2 mg, 12.7 µmol, 80 mol%, 4 mol% relative to $MeNH_2$)[4] was weighed into a ½ dram vial, removed from the glovebox and dissolved in MeCN-D_3 (160 µL). To the solution of cage **4** was then added $MeNH_2$ as a 2M THF solution (160 µL, 0.318 mmol, 20 equiv), followed by the MeCN solution of $Sc(OTf)_3$. The mixture was stirred vigorously for 5 h at RT. *Note: some cloudiness was observed in the reaction mixture.* After stirring for 5 h, a 100 µL aliquot was removed from the reaction mixture, diluted with 500 µL $CDCl_3$ and analyzed by ^{1}H NMR and MALDI-MS. The reaction mixture was then transferred to a 20 mL vial with $CDCl_3$ and concentrated in vacuo. The solid was dried under high-vacuum for 1 h to remove residual $MeNH_2$.

Cage Reassembly: The residue was re-dissolved in 4.8 mL of $CDCl_3$ and 0.52 mL of MeCN-D_3 (3 mM total) (*Note: some cloudiness was observed)* and 200 mg of powdered 5 Å molecular sieves were added. The solution was stirred overnight at RT (ca. 17 hours), at which point a 150 µL aliquot was filtered through a small plug of neutral alumina, concentrated to dryness, diluted with 600 µL of $CDCl_3$ and analyzed by ^{1}H NMR and MALDI-MS.

^{1}H NMR and MALDI-MS Analysis of Cage Disassembly/Reassembly

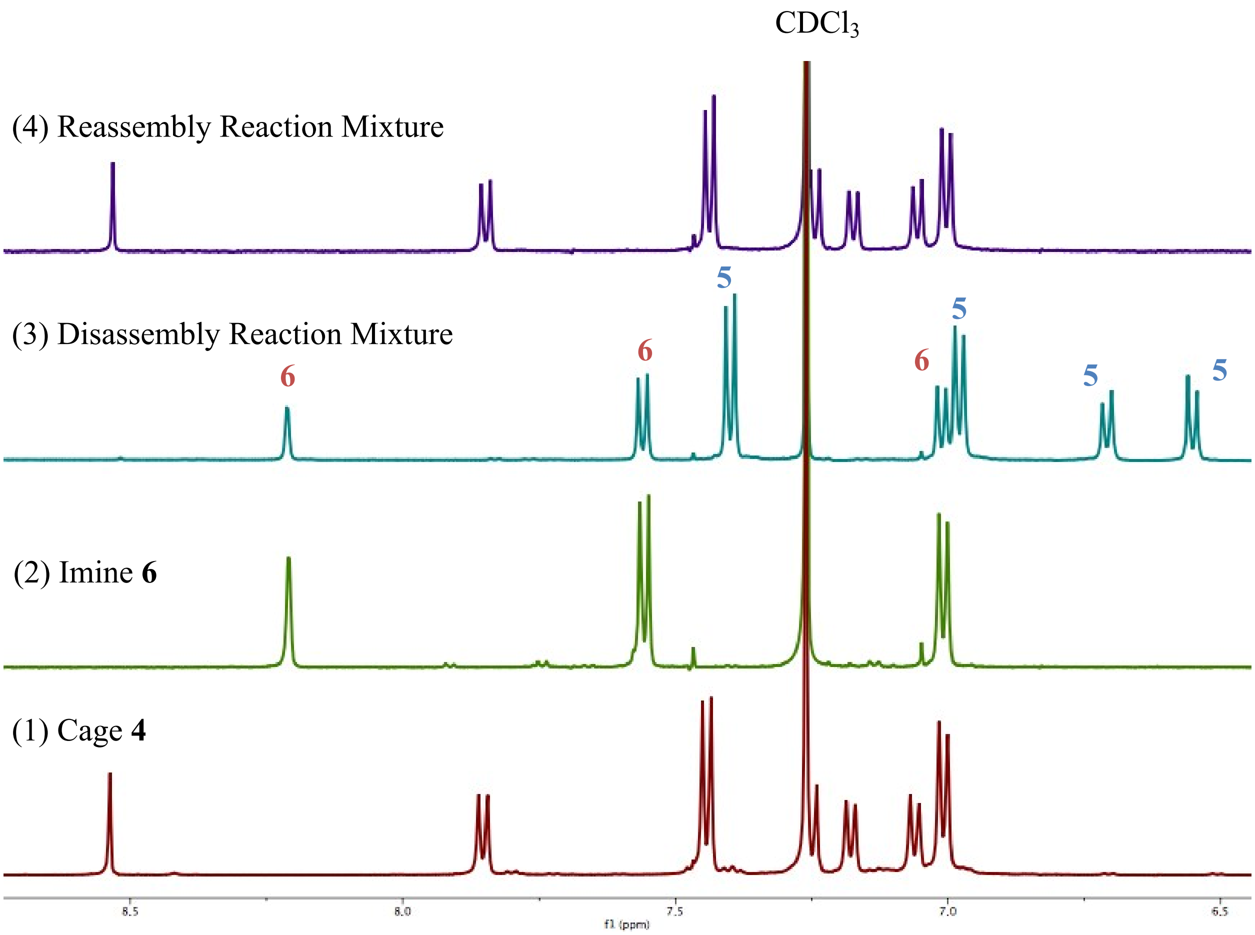

Figure S4. Expanded aromatic region of ^{1}H NMR spectra of cage disassembly/reassembly experiments. (1) ^{1}H NMR of Cage **4**. (2) ^{1}H NMR of independently synthesized imine **6**; NMR shown was recorded in a solvent mixture of $CDCl_3$/THF-D_8/MeCN-D_3 in similar proportions to that used in disassembly reaction conditions. (3) ^{1}H NMR analysis of the disassembly reaction mixture. Comparison to authentic standard allows for assignment of **6**. The remaining peaks have been assigned to macrocycle **5**. (4) ^{1}H NMR analysis of reassembly reaction mixture. The sample was filtered through a small plug of neutral alumina and concentrated to dryness before analysis.

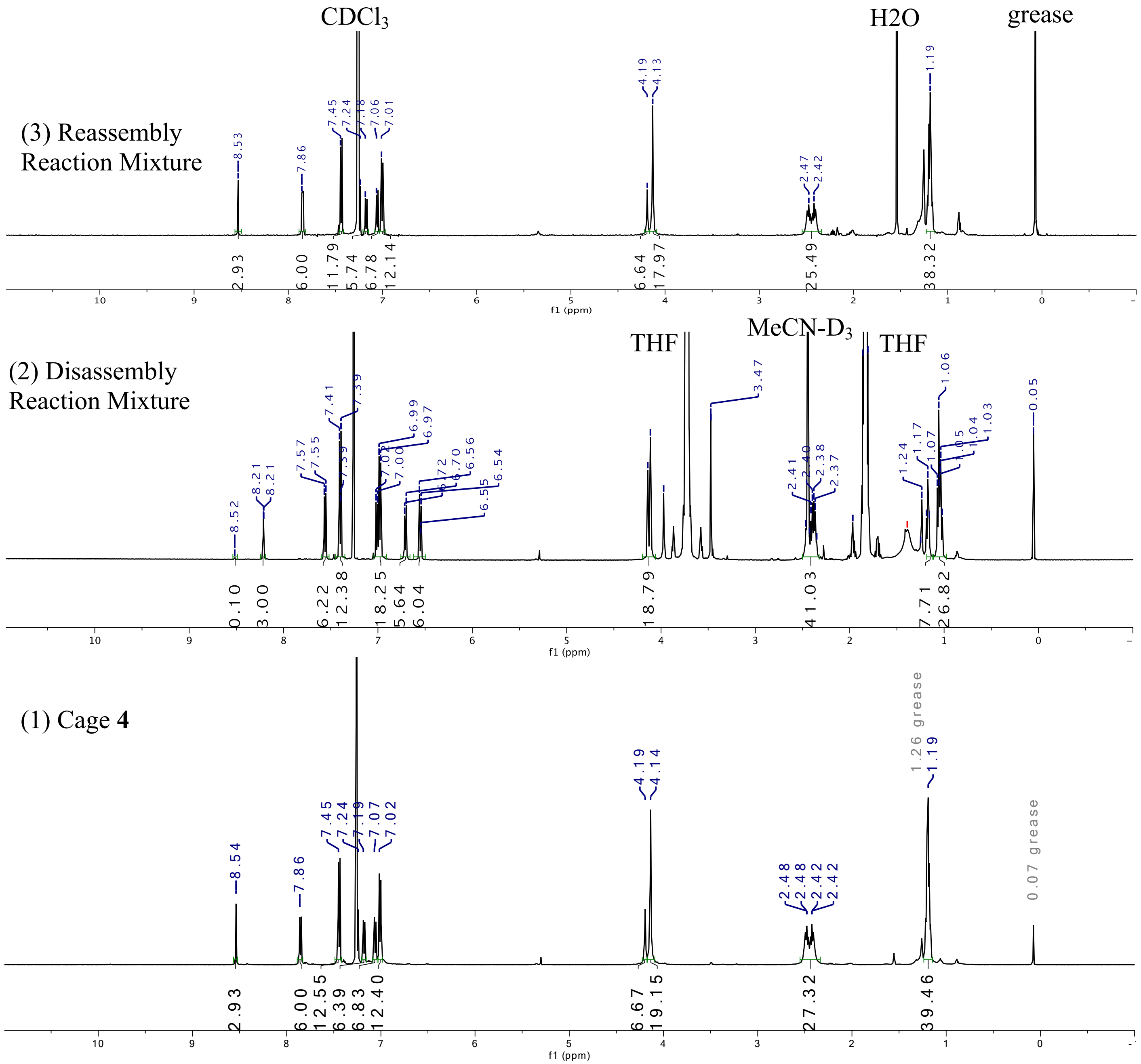

Figure S5. ^{1}H NMR spectra of disassembly/reassembly. Spectra were zoomed-in for clarity. Mestrenova GSD integration mode used for integration of the disassembly mixture spectrum.

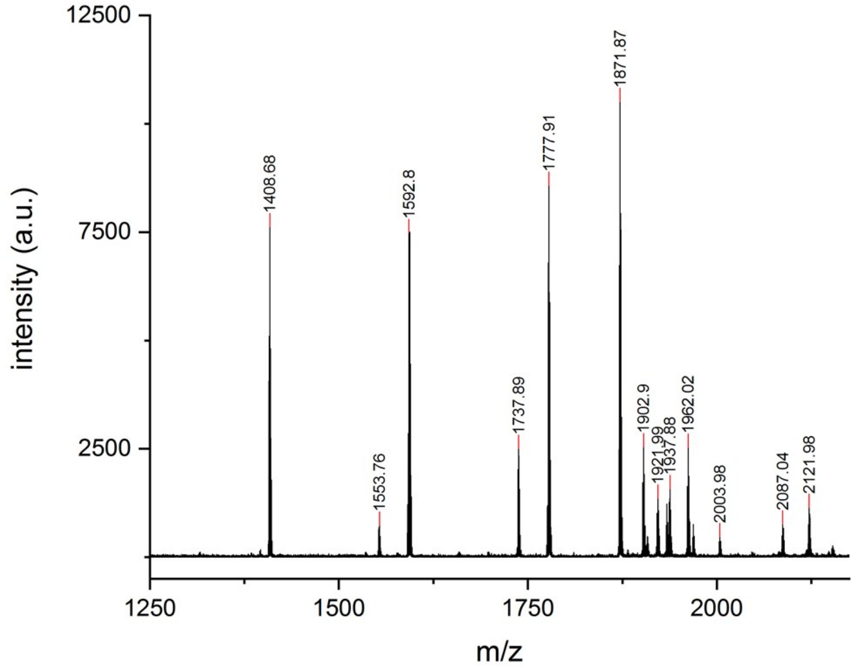

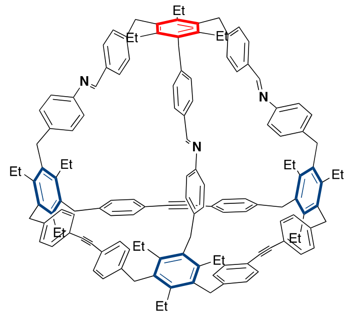

Chemical Formula: $C_{141}H_{135}N_3$
Exact Mass: 1870.066
Molecular Weight: 1871.652

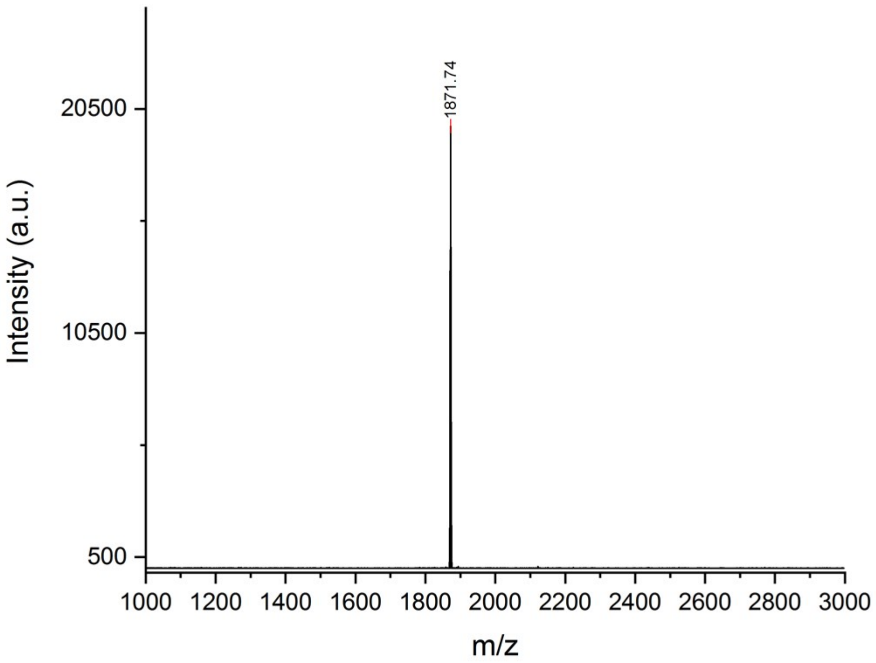

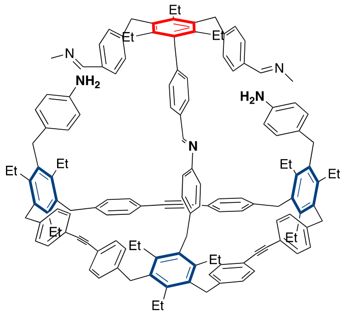

Chemical Formula: $C_{143}H_{145}N_5$
Exact Mass: 1932.150
Molecular Weight: 1933.768

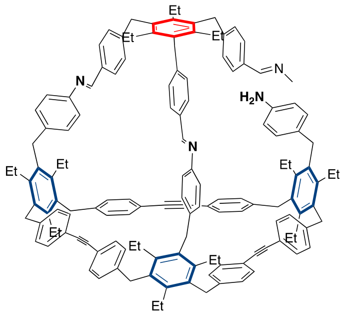

Chemical Formula: $C_{142}H_{140}N_4$
Exact Mass: 1901.108
Molecular Weight: 1902.710

Figure S6. MALDI-MS analysis of the disassembly (top) using $MeNH_2/Sc(OTf)_3$ and of the reassembly reaction mixture (bottom). MALDI analysis of the disassembly reaction mixture indicates the presence of partially disassembled cage species and remaining cage. Predicted masses for reaction components are displayed to the right for reference

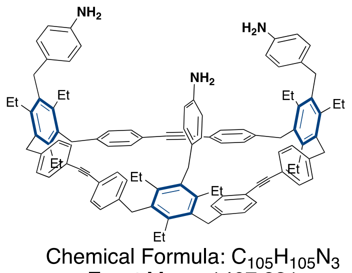

Chemical Formula: $C_{105}H_{105}N_3$
Exact Mass: 1407.831
Molecular Weight: 1409.016

B. Cage Disassembly Using Trifluoroacetic Acid (TFA)

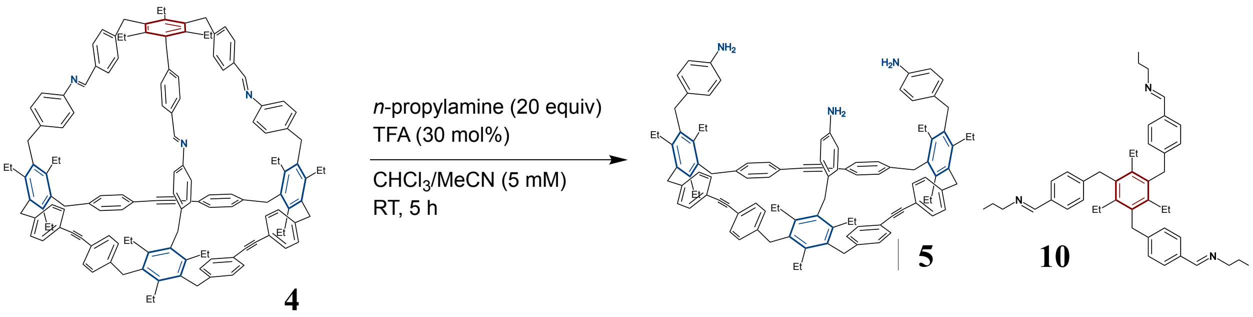

In a 20 mL reaction vial, cage **4** (16.9 mg, 9 μmol, 1 equiv) was dissolved in $CDCl_3$ (2 mL, 5 mM) and stirred until dissolved. To the solution was then added n-propylamine (15 μL, 0.18 mmol, 20 equiv), followed by TFA (0.2 μL, 2.7 μmol, 30 mol%). The mixture was then stirred vigorously at room temperature for 5 hours. After 5 h, a 50 μL aliquot was removed, diluted with 0.5 mL $CDCl_3$, and analyzed by ^1H NMR.

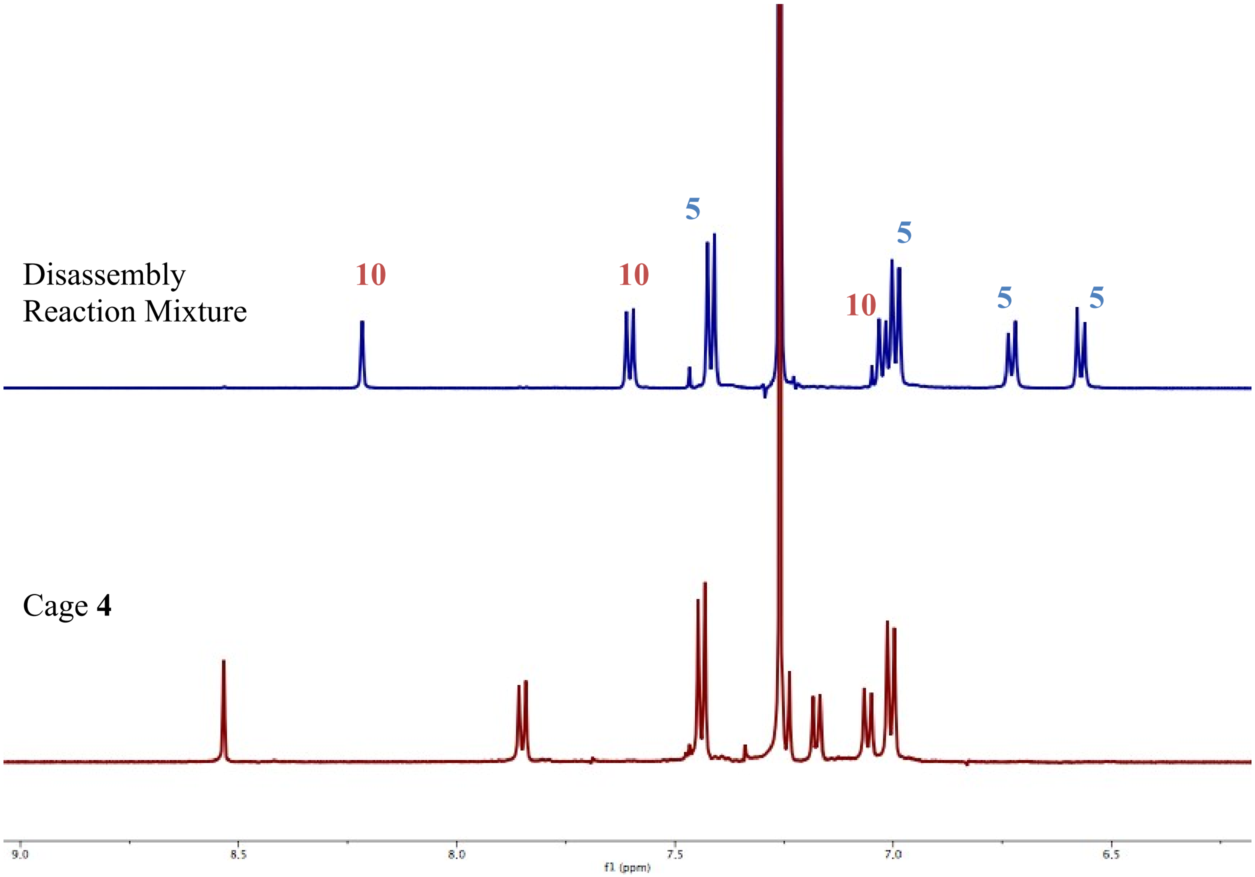

Figure S7. Expanded aromatic region of the ^1H NMR spectra of cage **4** (bottom) and disassembly reaction mixture using TFA (top). Peaks corresponding to macrocycle **5** and tris-imine **7** have been assigned consistently with Figure S5.

S.4 ^{1}H and ^{13}C NMR Spectra

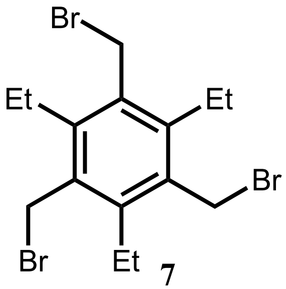

7

(^{1}H NMR, $CDCl_3$, 500 MHz)

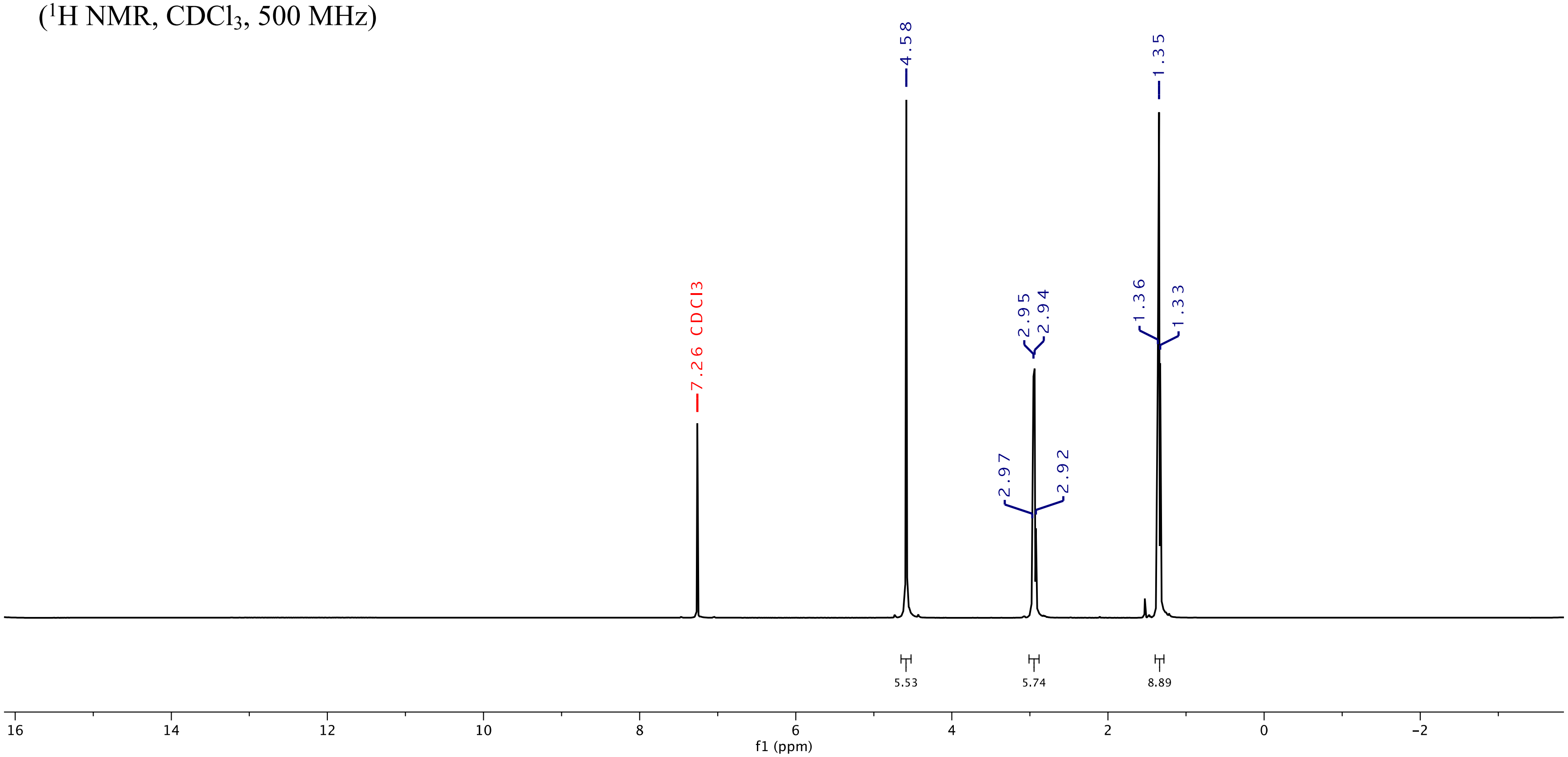

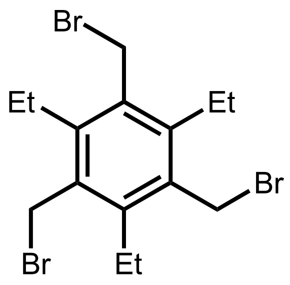

7

(^{13}C NMR, $CDCl_3$, 126 MHz)

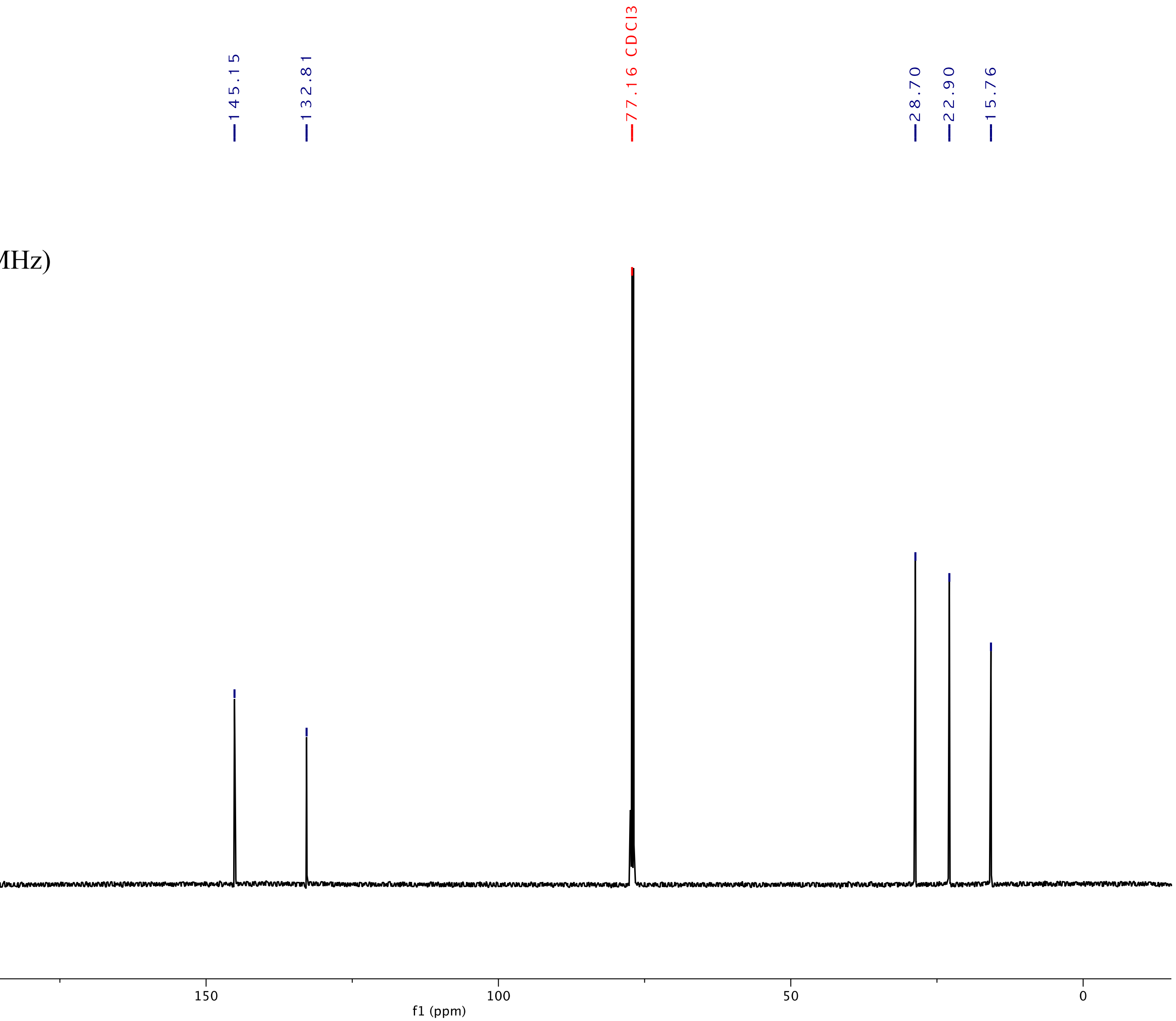

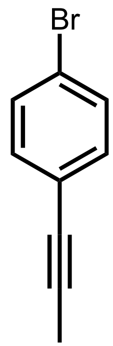

8

(^{1}H NMR, $CDCl_3$, 500 MHz)

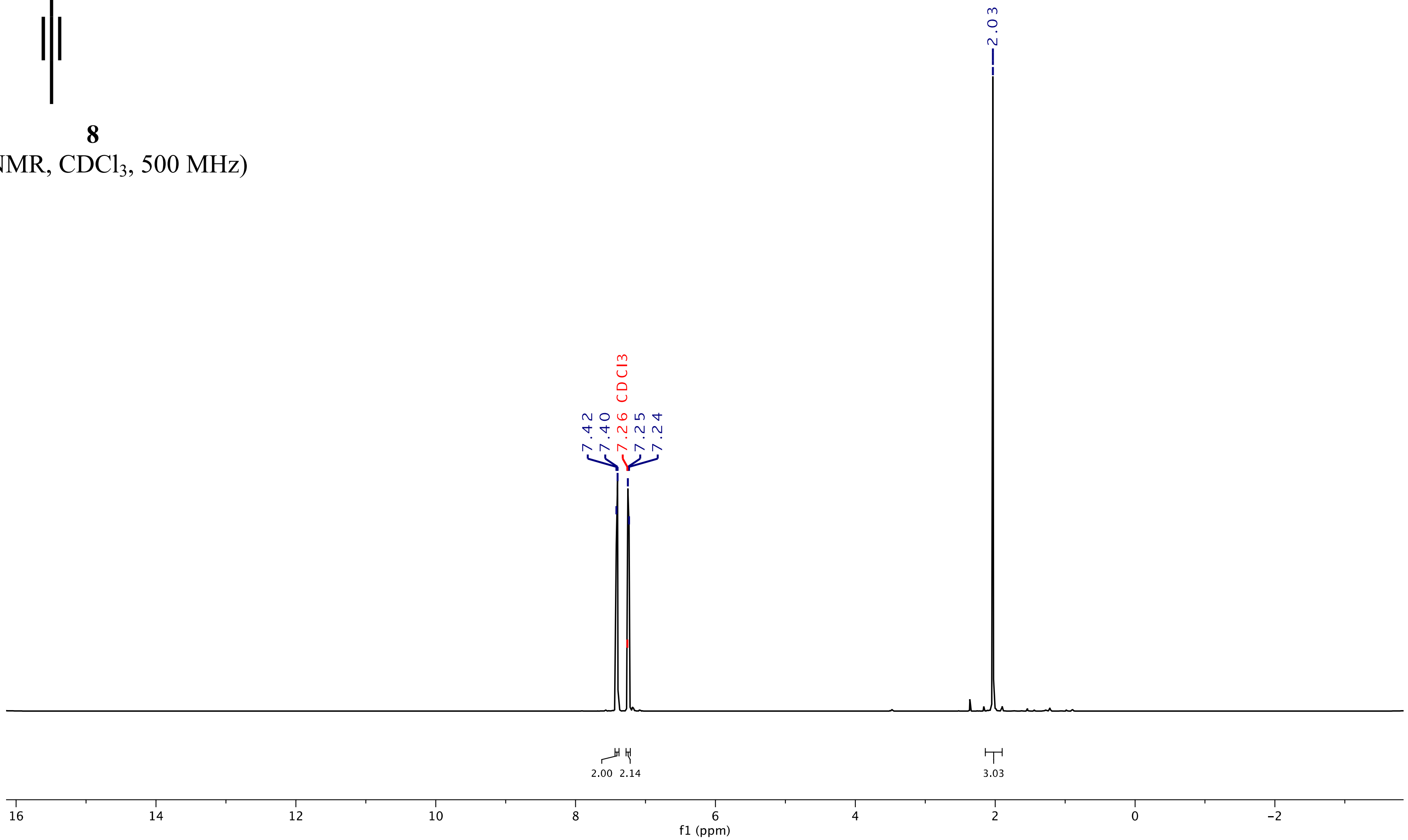

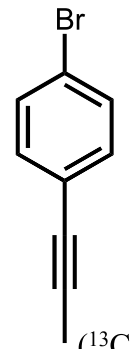

8

(^{13}C NMR, $CDCl_3$, 126 MHz)

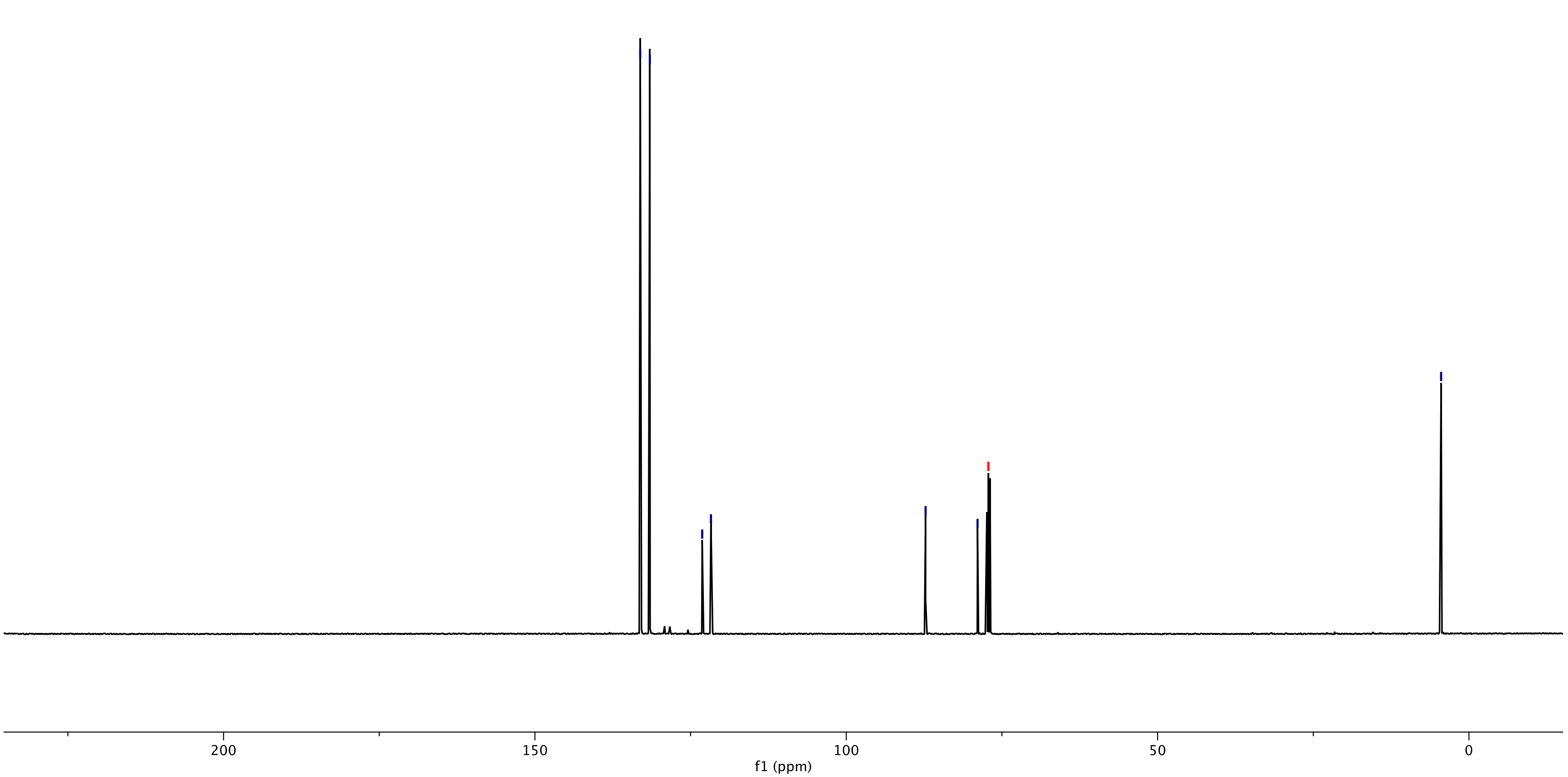

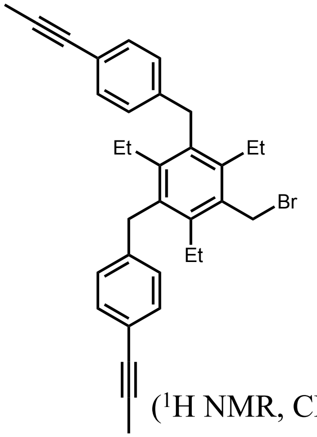

9
(^{1}H NMR, $CDCl_3$, 500 MHz)

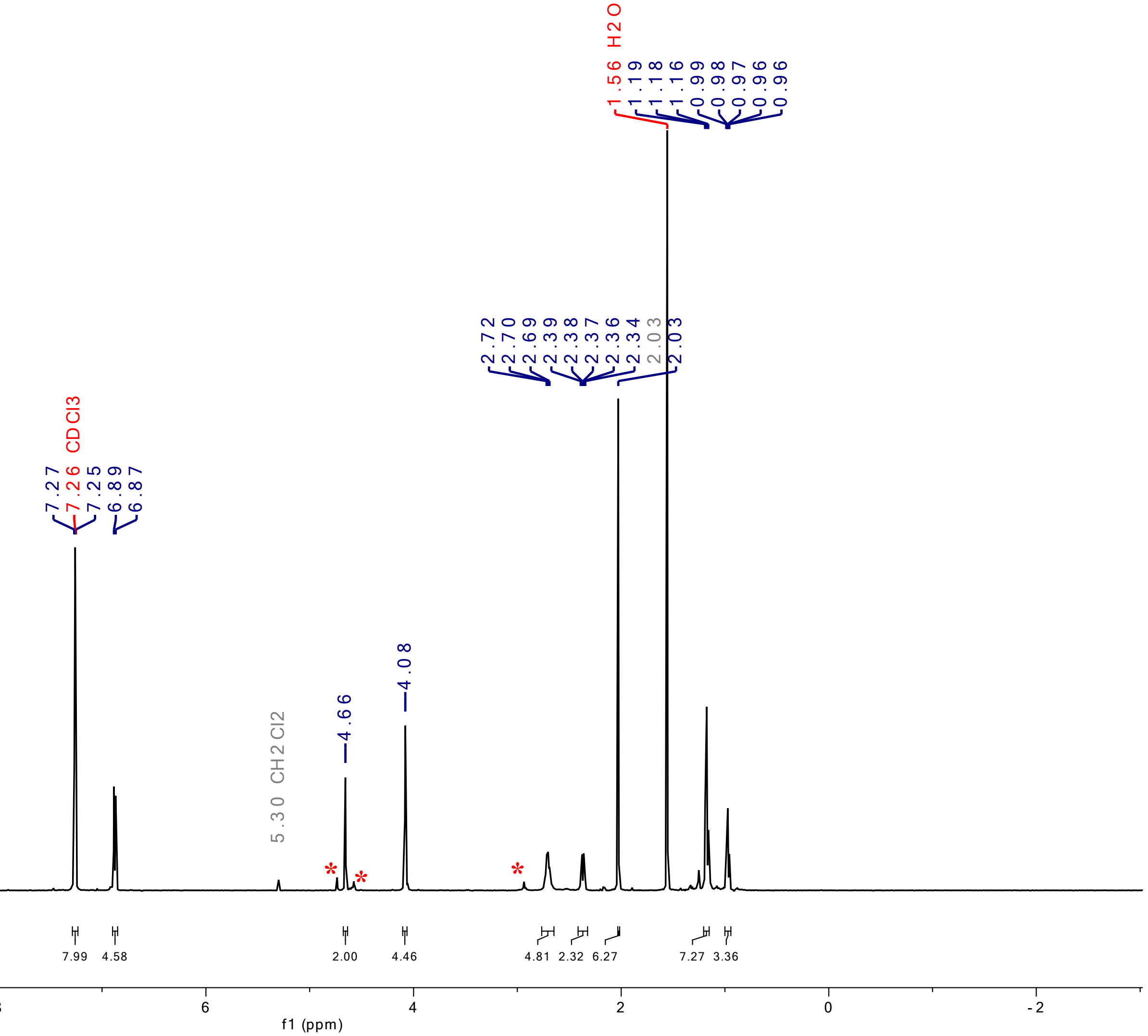

Note: compound **9** coelutes with an unknown impurity (*) which may be an isomer.

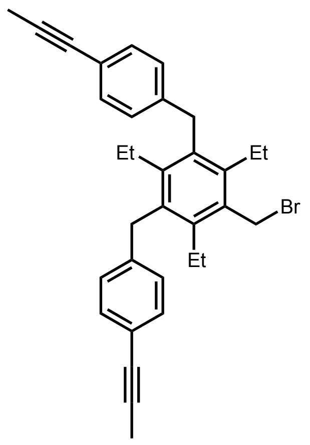

9
(^{13}C NMR, $CDCl_3$, 126 MHz)

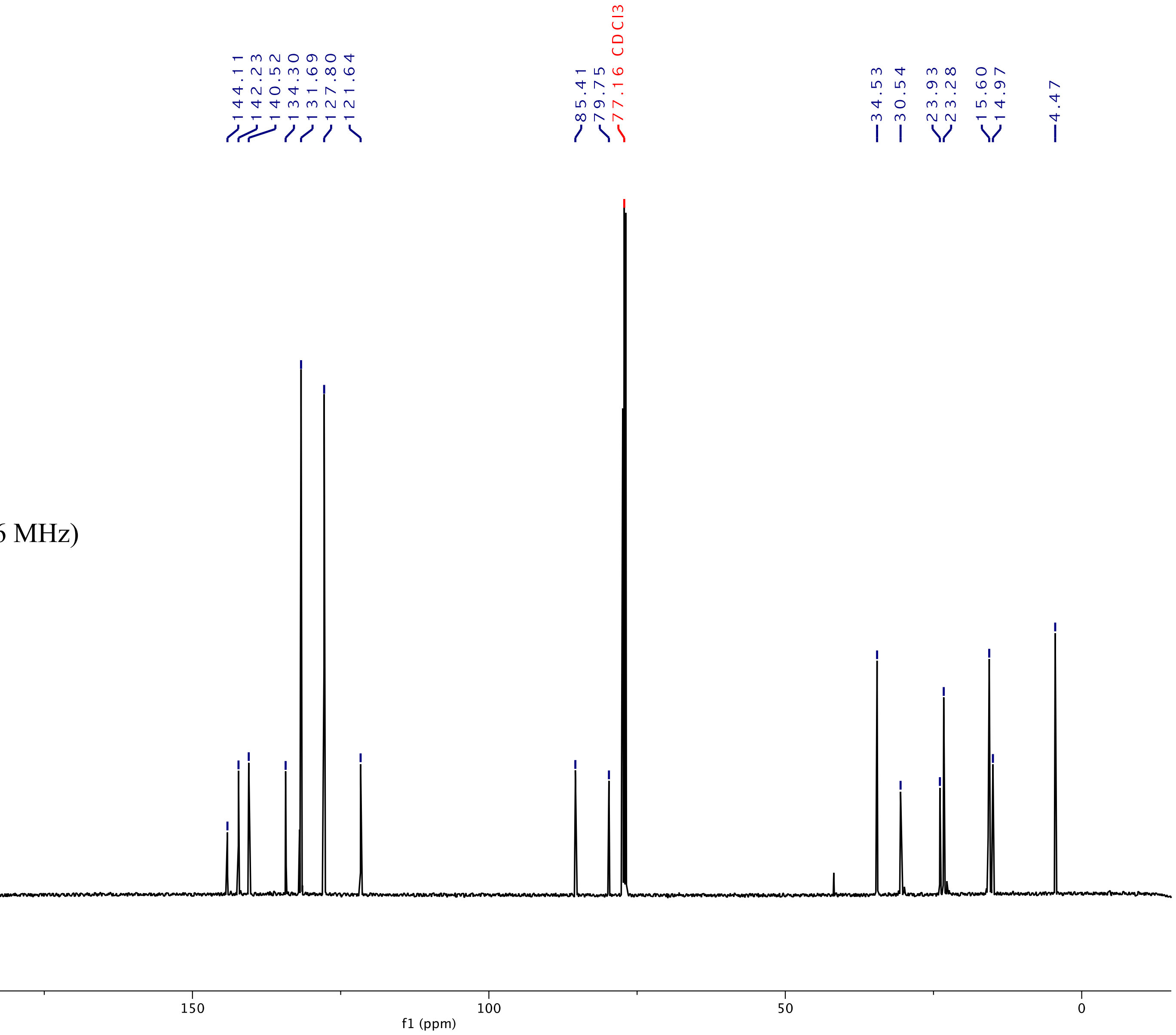

Note: compound **9** coelutes with an unknown impurity which may be an isomer.

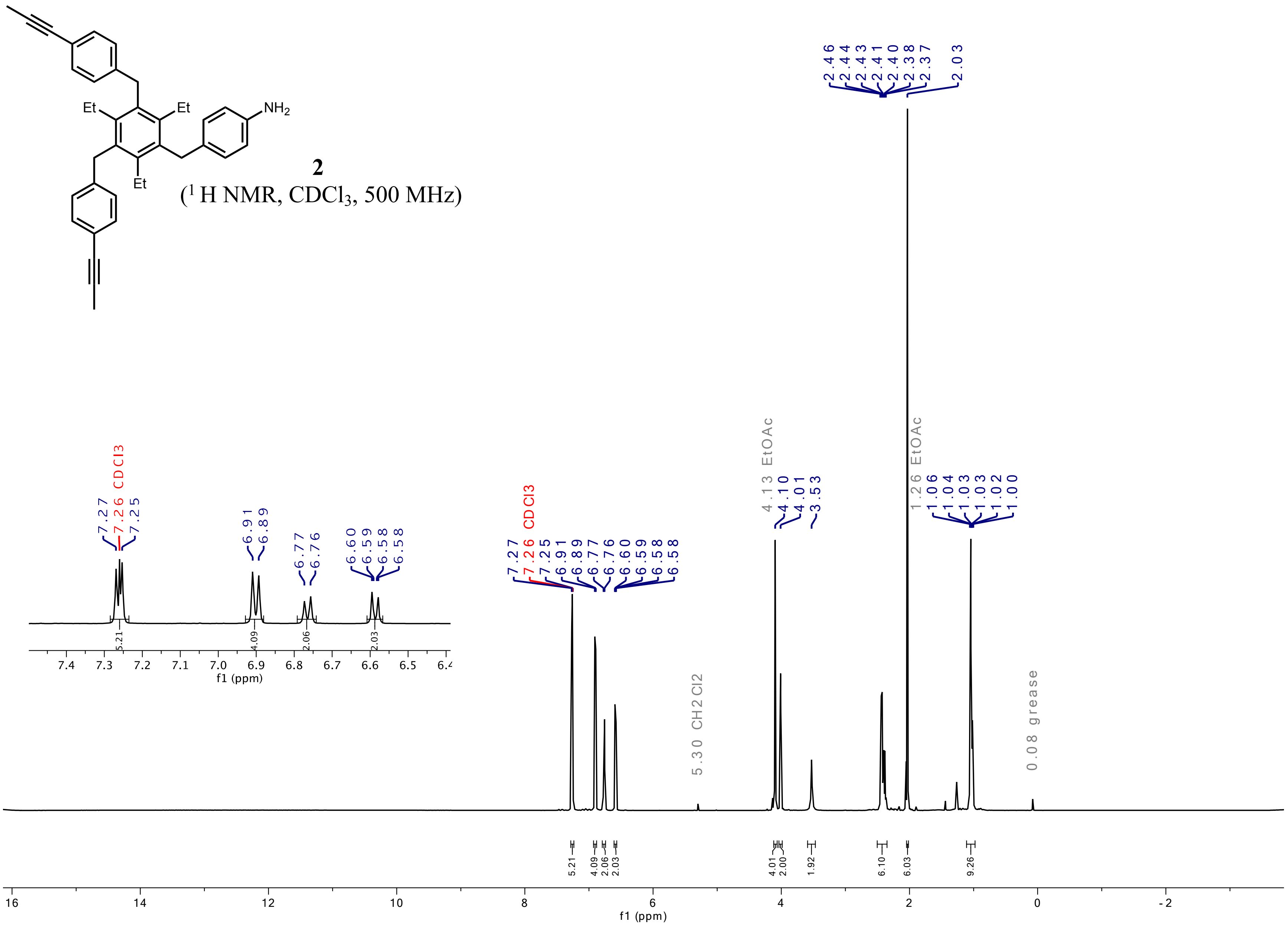

Et
Et
NH2
Et
2
(1 H NMR, CDCl3, 500 MHz)
7.27
7.26 CDCl3
7.25
6.91
6.89
6.77
6.76
6.60
6.59
6.58
6.58
5.21
4.09
2.06
2.03
f1 (ppm)
5.30 CH2Cl2
4.13 EtOAc
4.10
4.01
3.53
2.46
2.44
2.43
2.41
2.40
2.38
2.37
2.03
1.26 EtOAc
1.06
1.04
1.03
1.03
1.02
1.00
0.08 grease
4.01
2.00
1.92
6.10
6.03
9.26

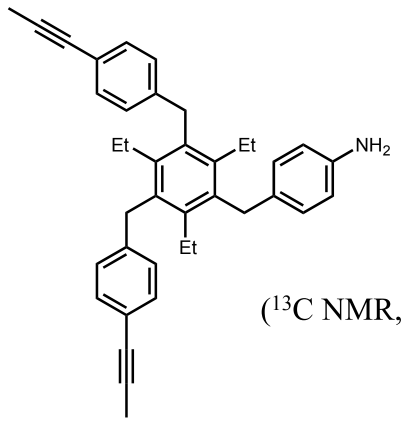

2
(^{13}C NMR, $CDCl_3$, 126 MHz)

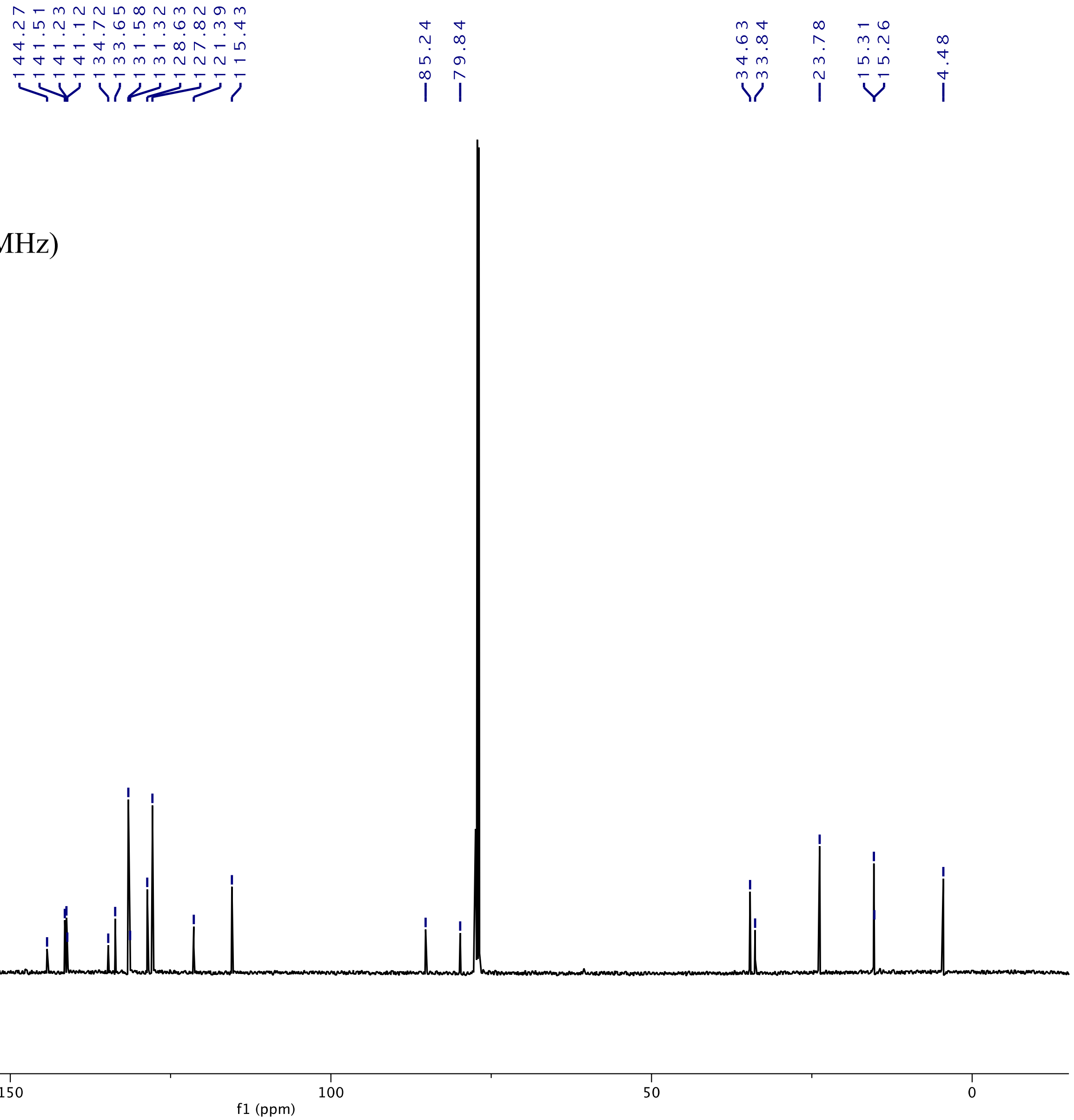

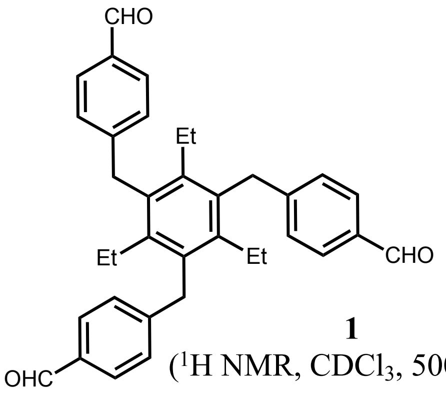

1

(^{1}H NMR, $CDCl_3$, 500 MHz)

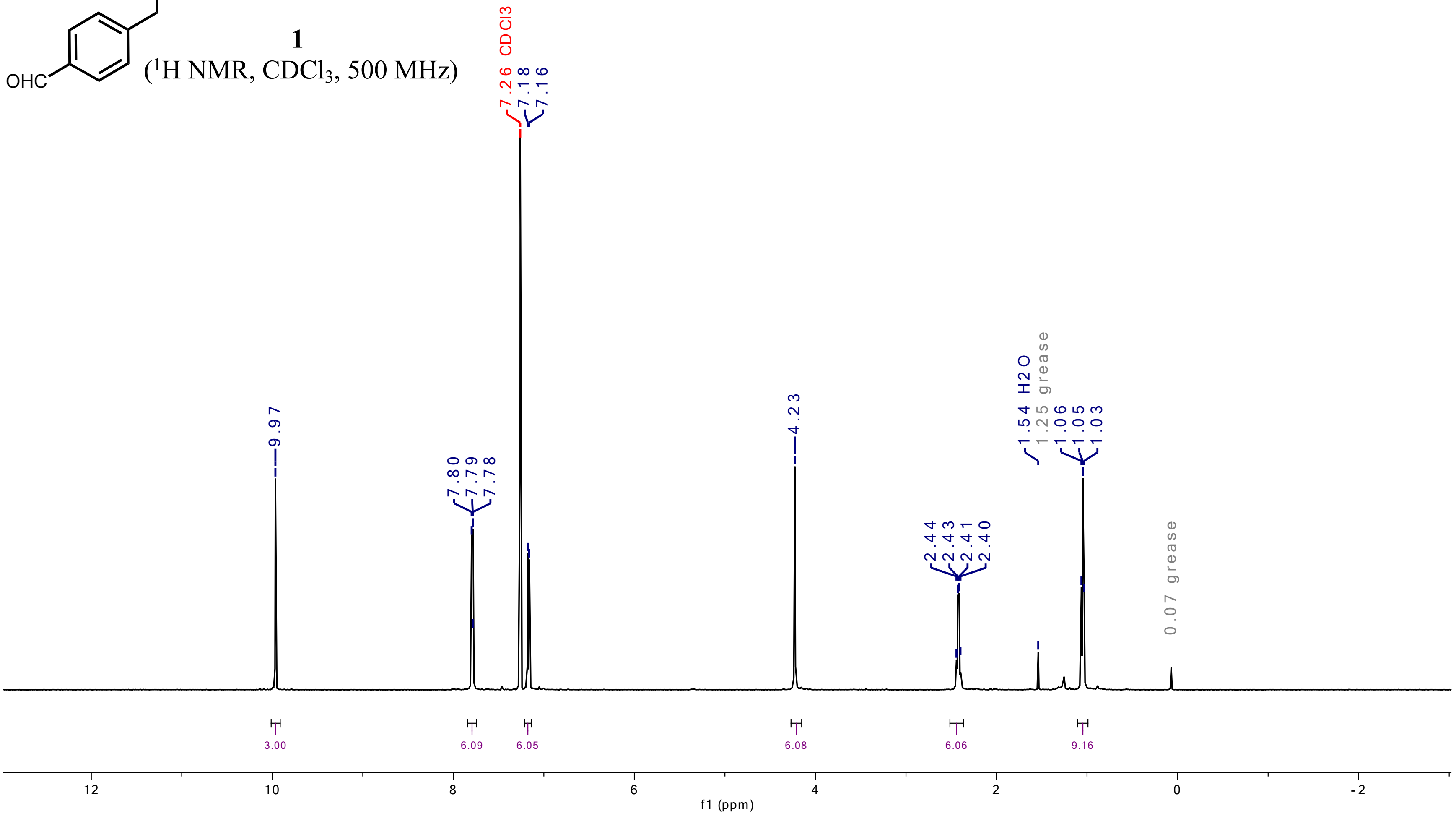

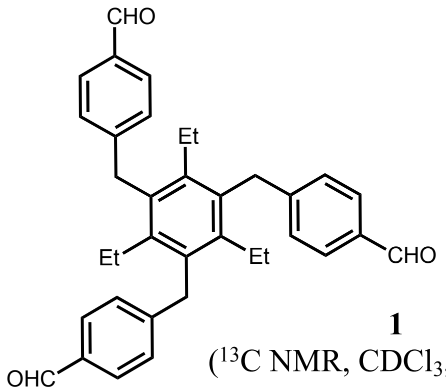

1
(^{13}C NMR, $CDCl_3$, 126 MHz)

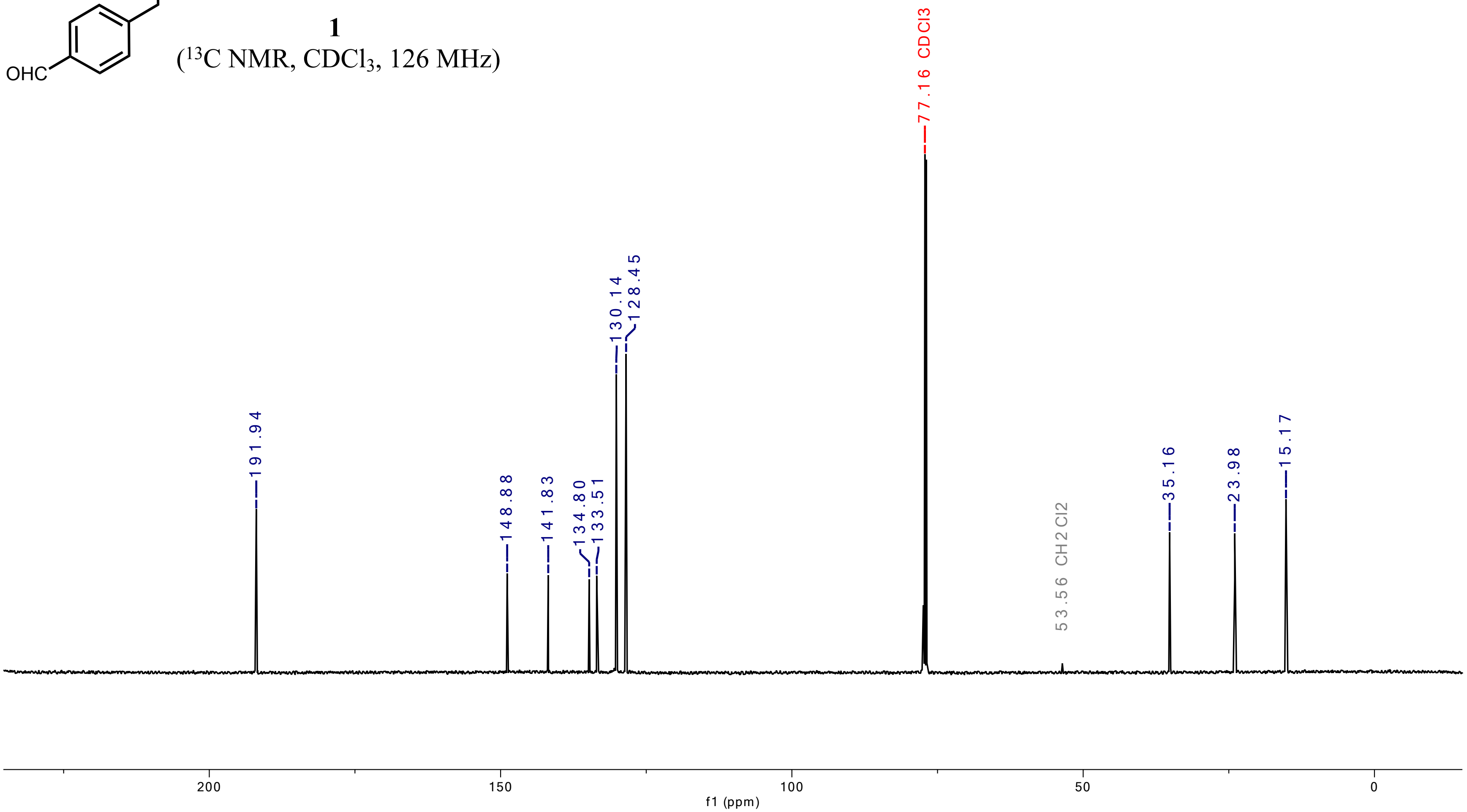

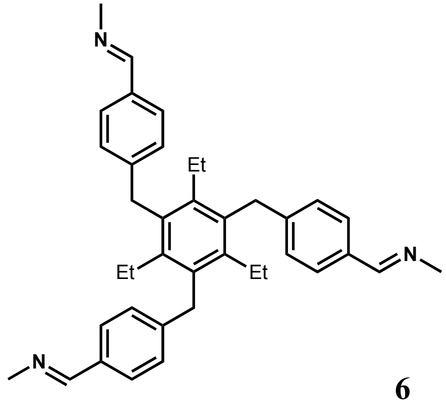

6

(^{1}H NMR, $CDCl_3$, 500 MHz)

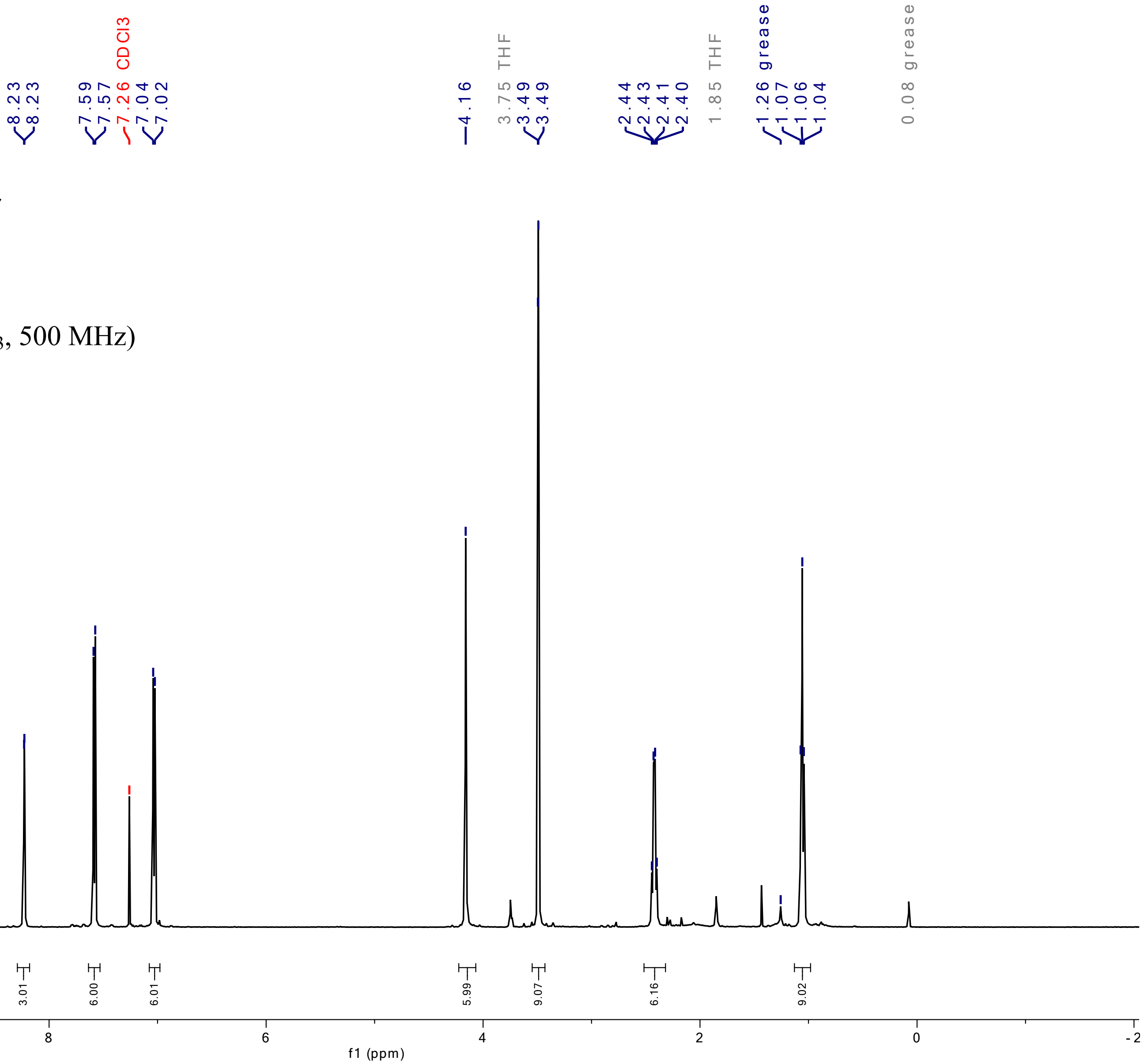

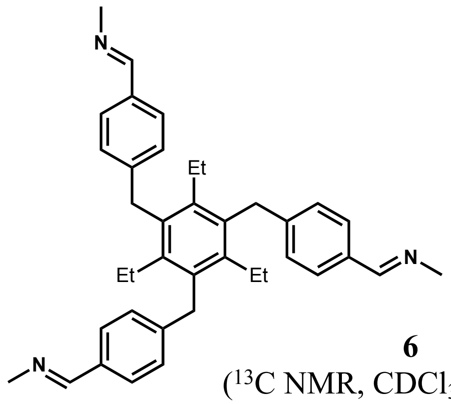

6

(^{13}C NMR, $CDCl_3$, 126 MHz)

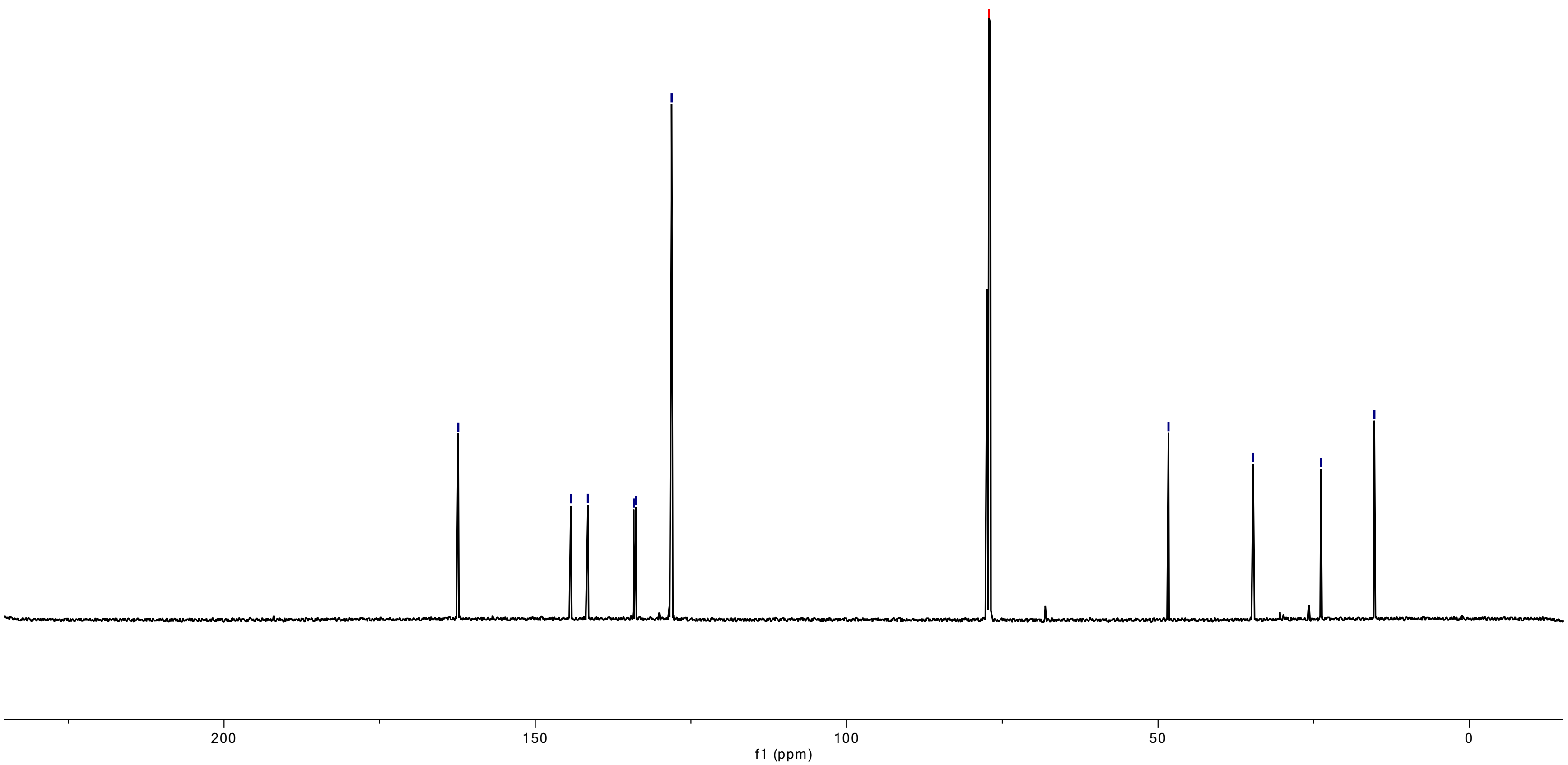

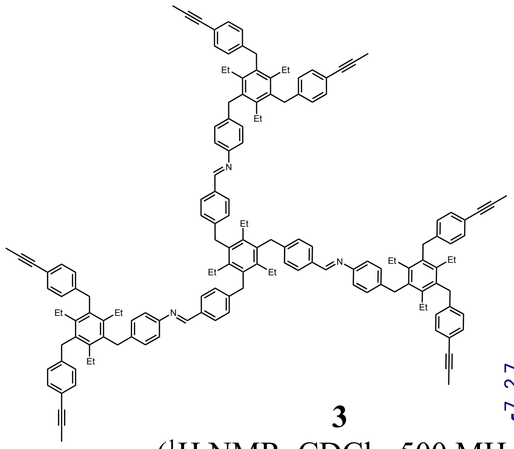

3

(^{1}H NMR, $CDCl_3$, 500 MHz)

*(Aromatic region of **3** in CD_2Cl_2)*

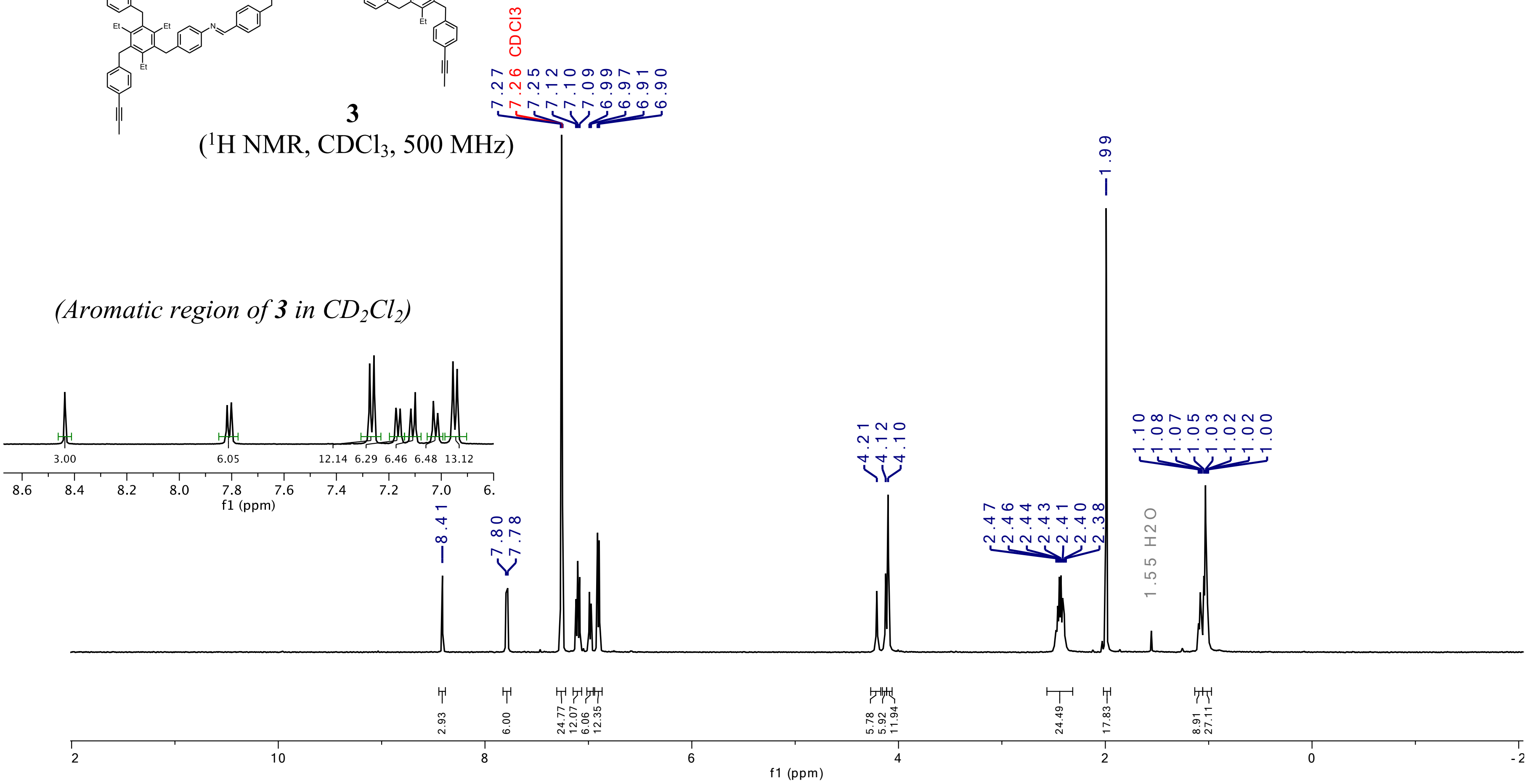

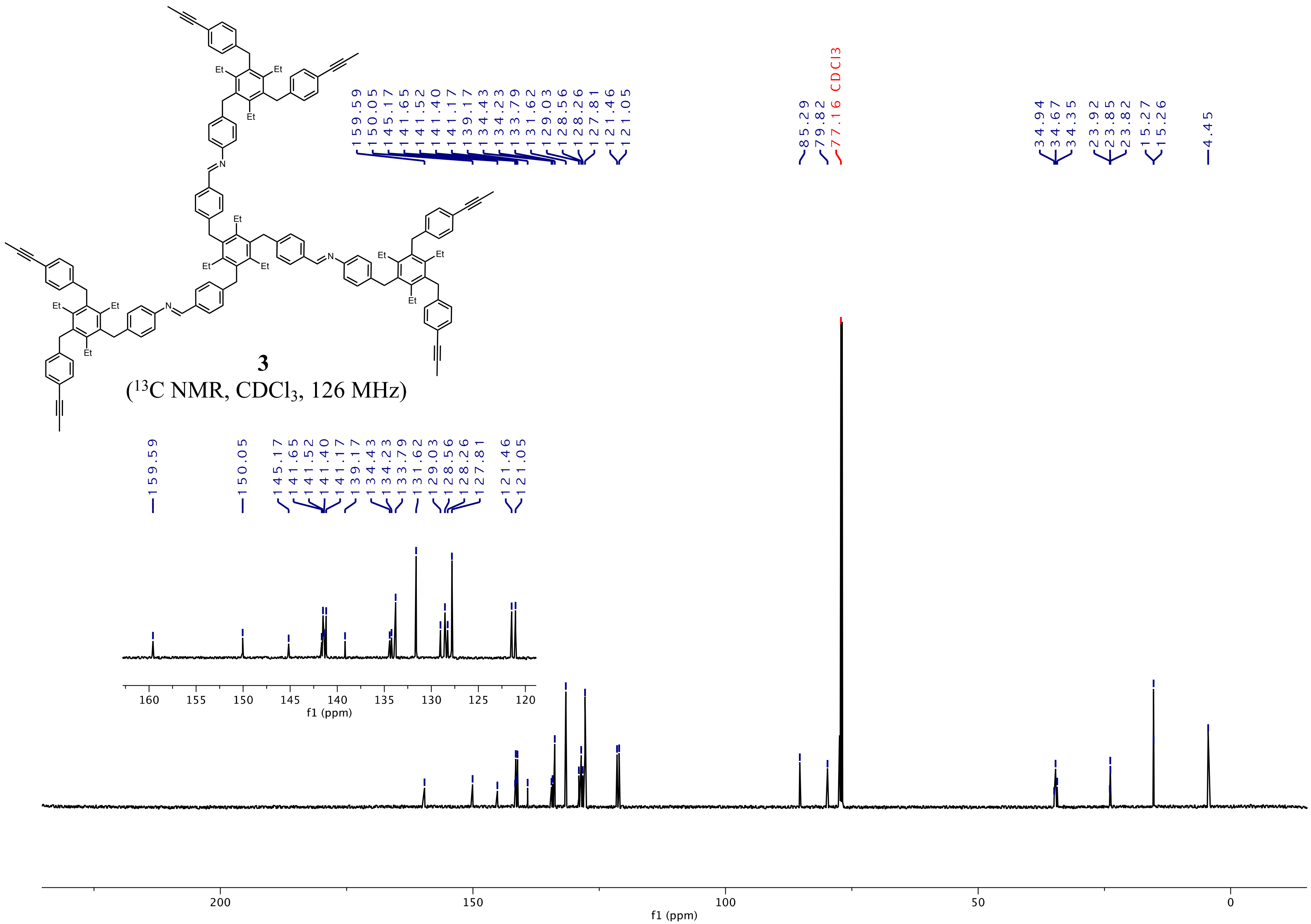

3

(^{13}C NMR, $CDCl_3$, 126 MHz)

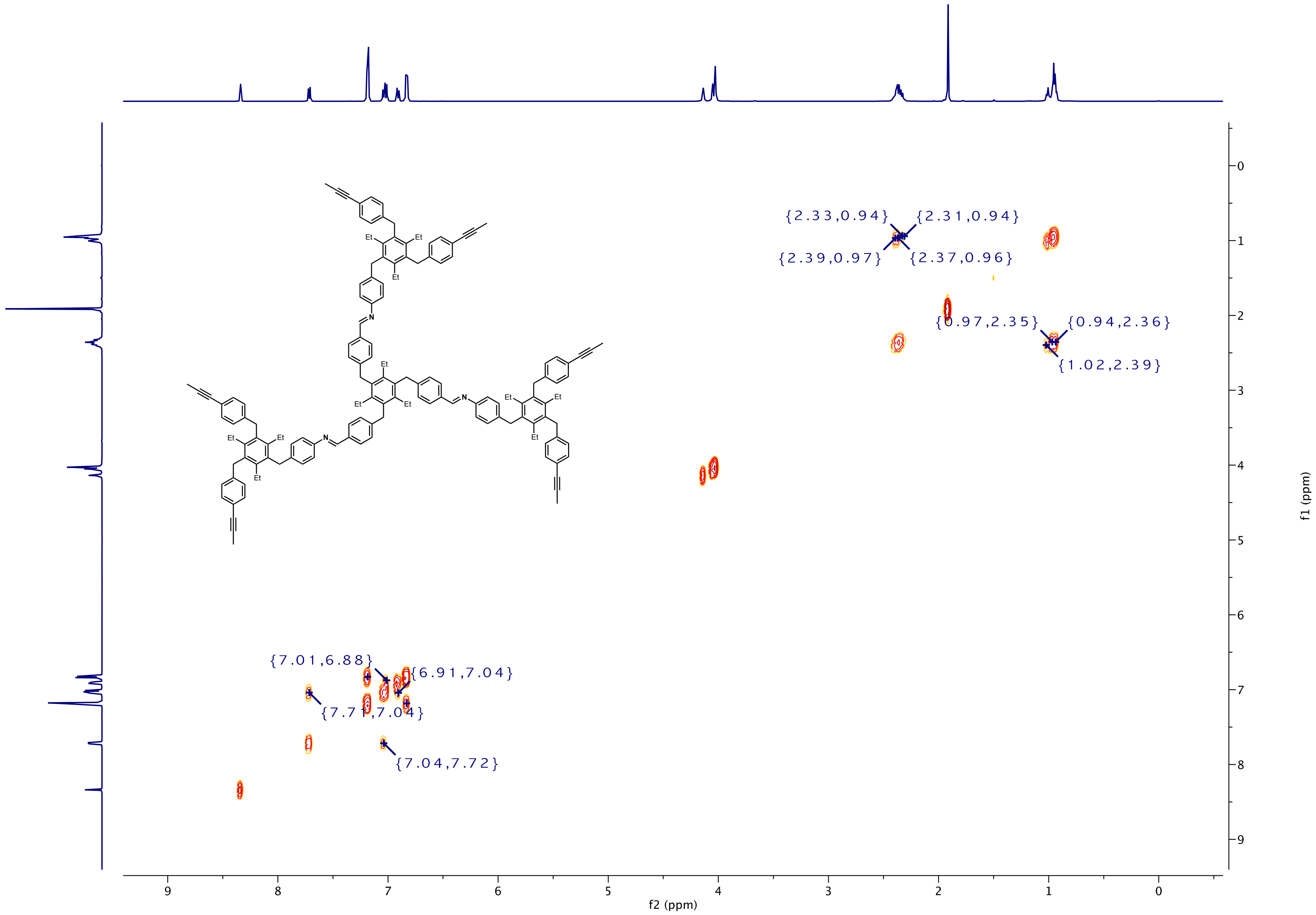

^{1}H-^{1}H COSY spectrum of precursor **3** (500 MHz, $CDCl_3$).

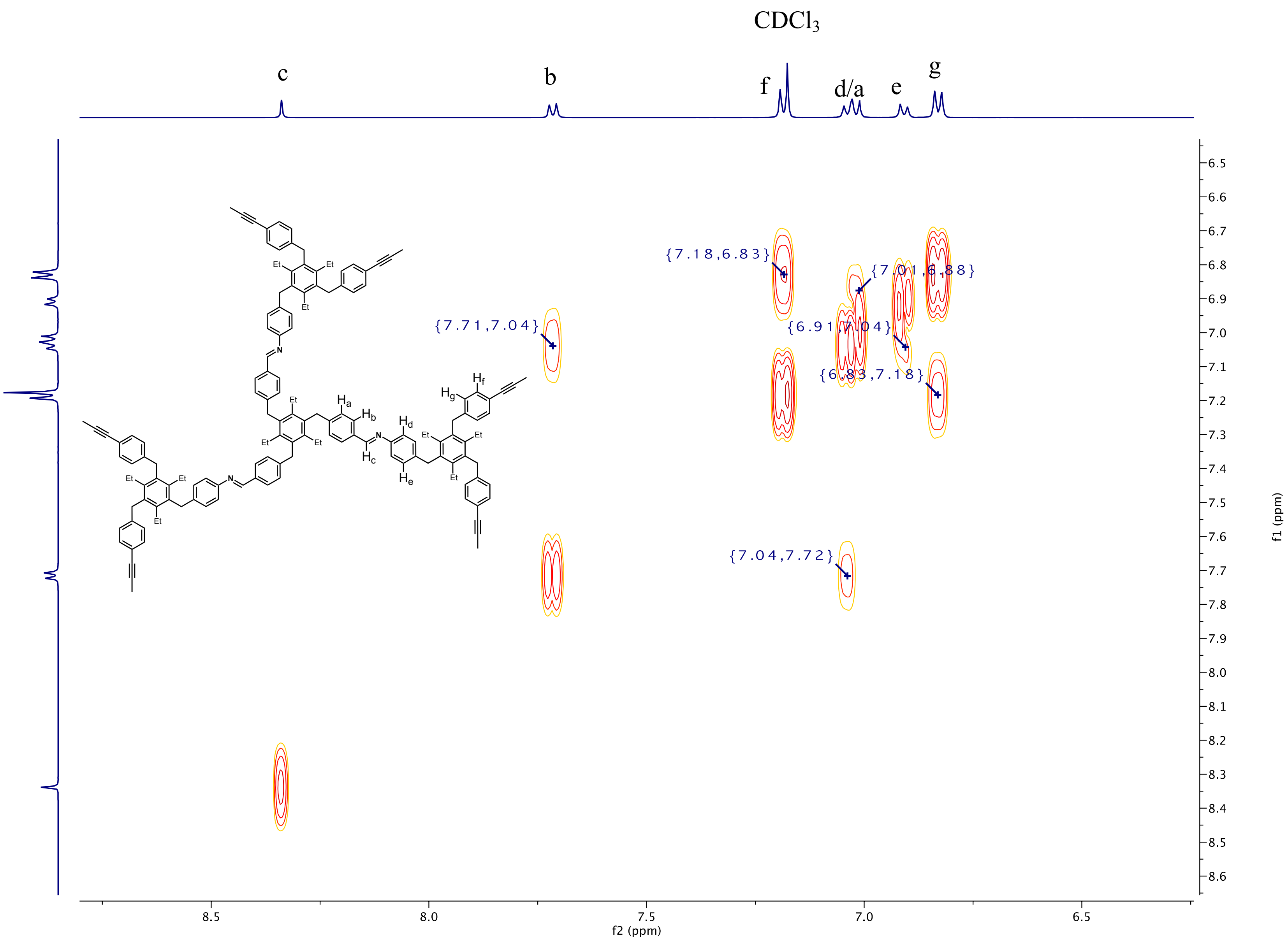

^{1}H-^{1}H COSY spectrum of precursor **3** (500 MHz, $CDCl_3$).

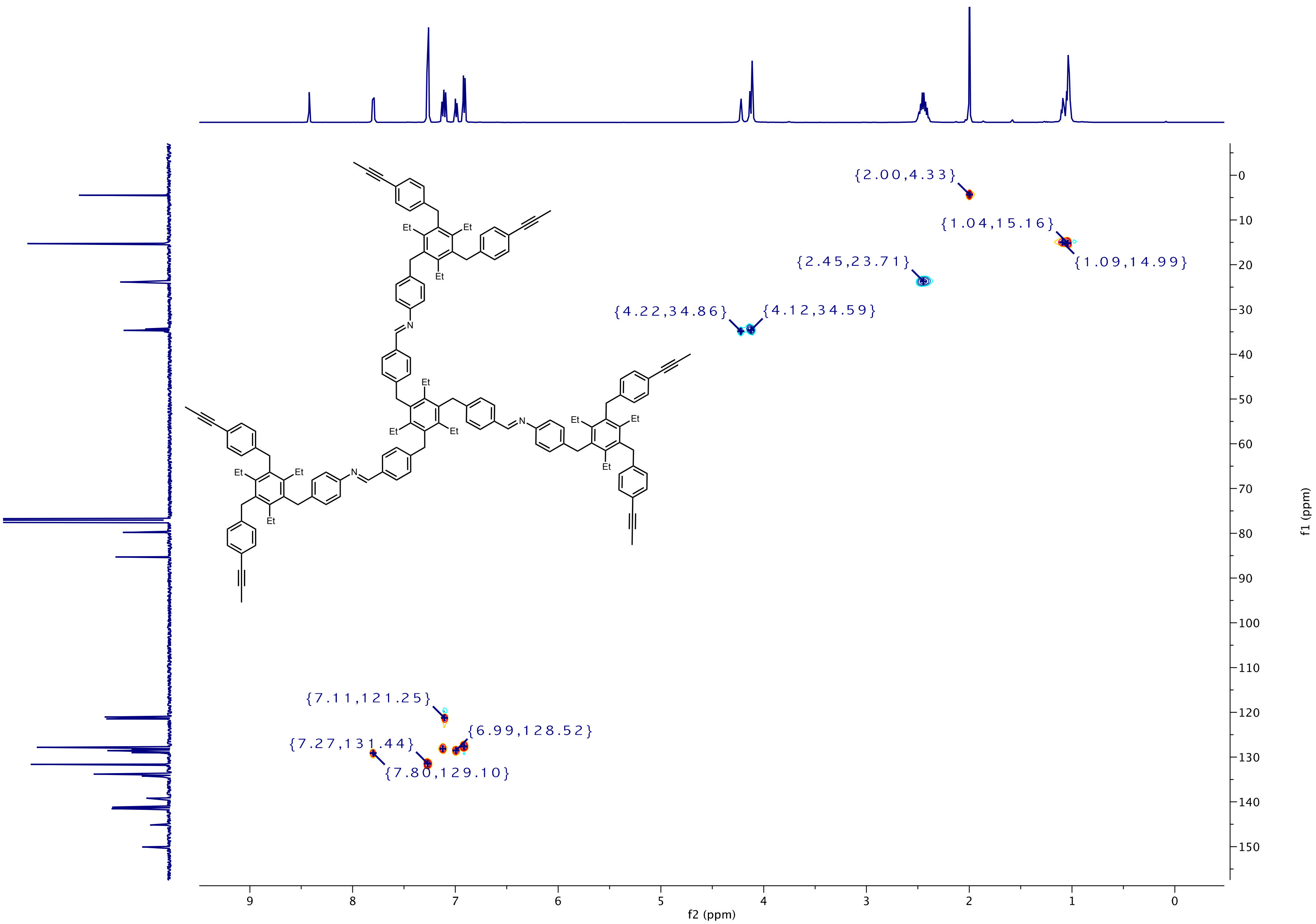

^{1}H-^{13}C HSQC Spectrum of precursor **3** ($CDCl_3$, 500 MHz).

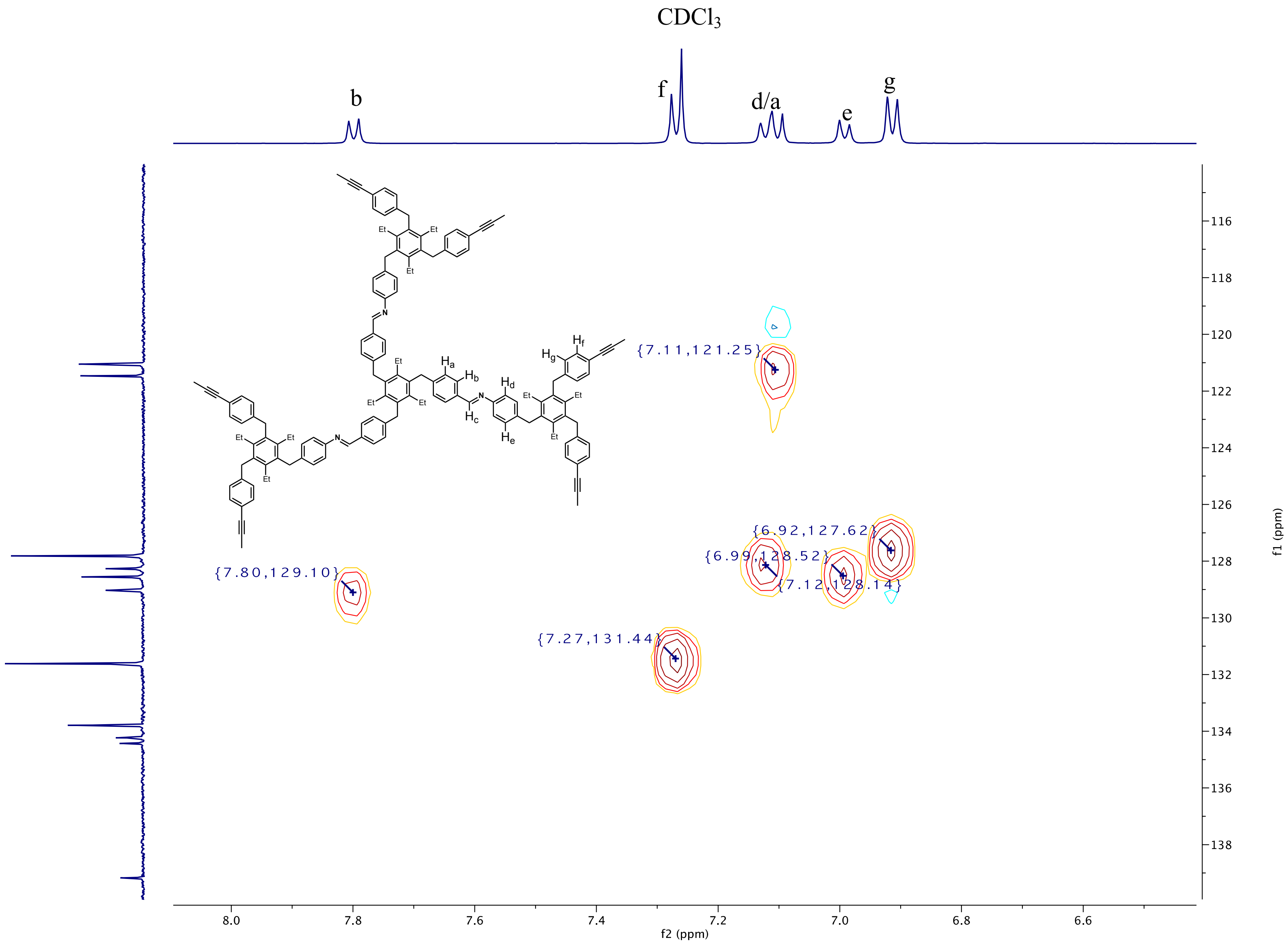

^{1}H-^{13}C HSQC Spectrum of precursor **3** ($CDCl_3$, 500 MHz). The crosspeak of H_c was outside the scan width of the experiment.

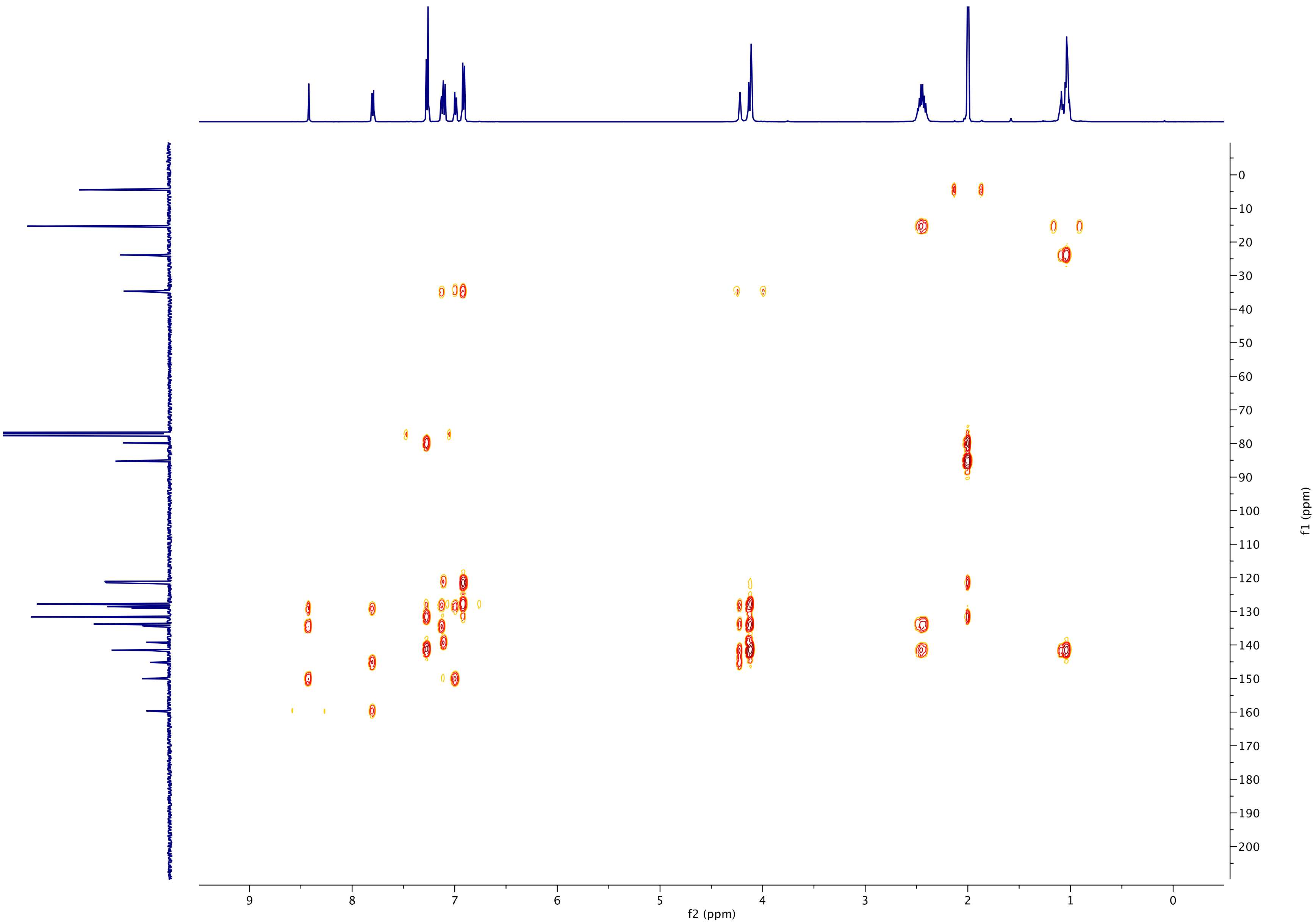

^{1}H-^{13}C HMBC of precursor **3** ($CDCl_3$, 500 MHz)

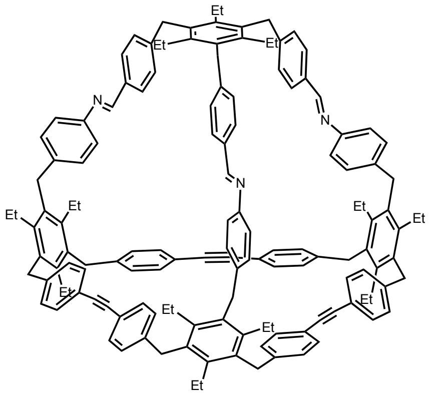

4

(^{1}H NMR, $CDCl_3$, 500 MHz)

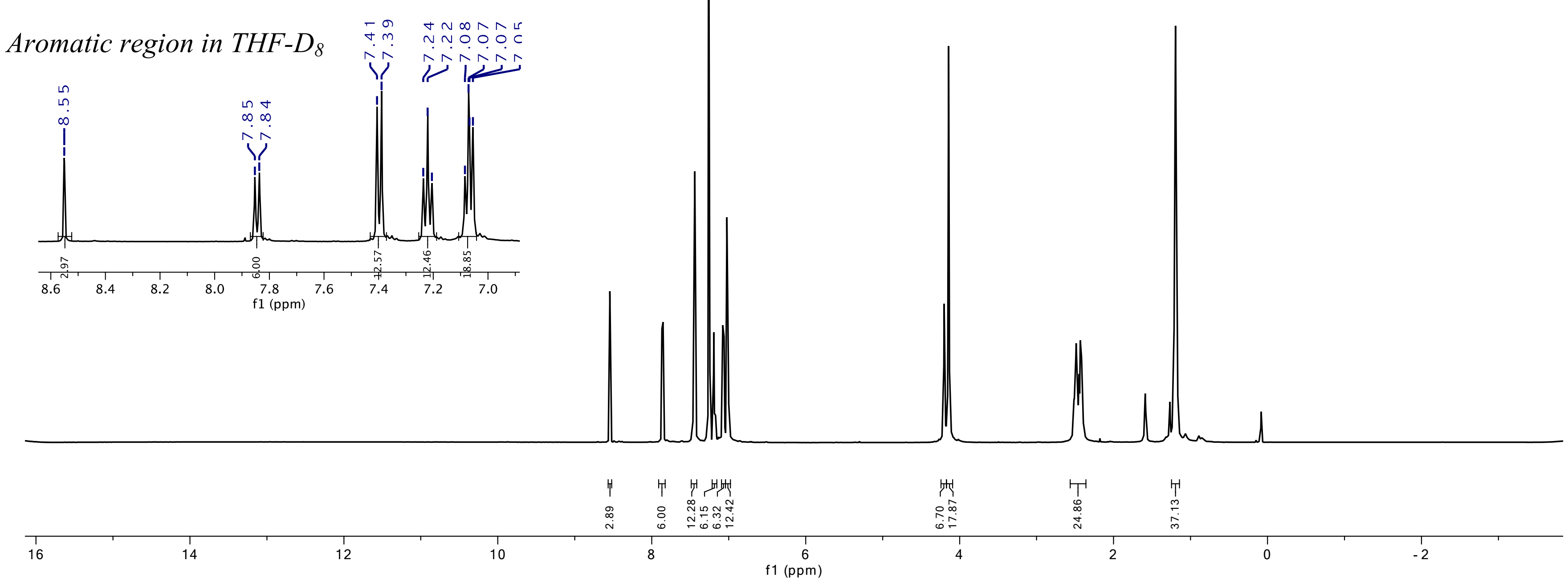

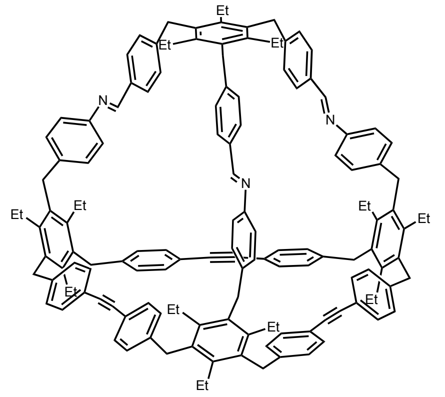

4
(^{13}C NMR, $CDCl_3$, 126 MHz)

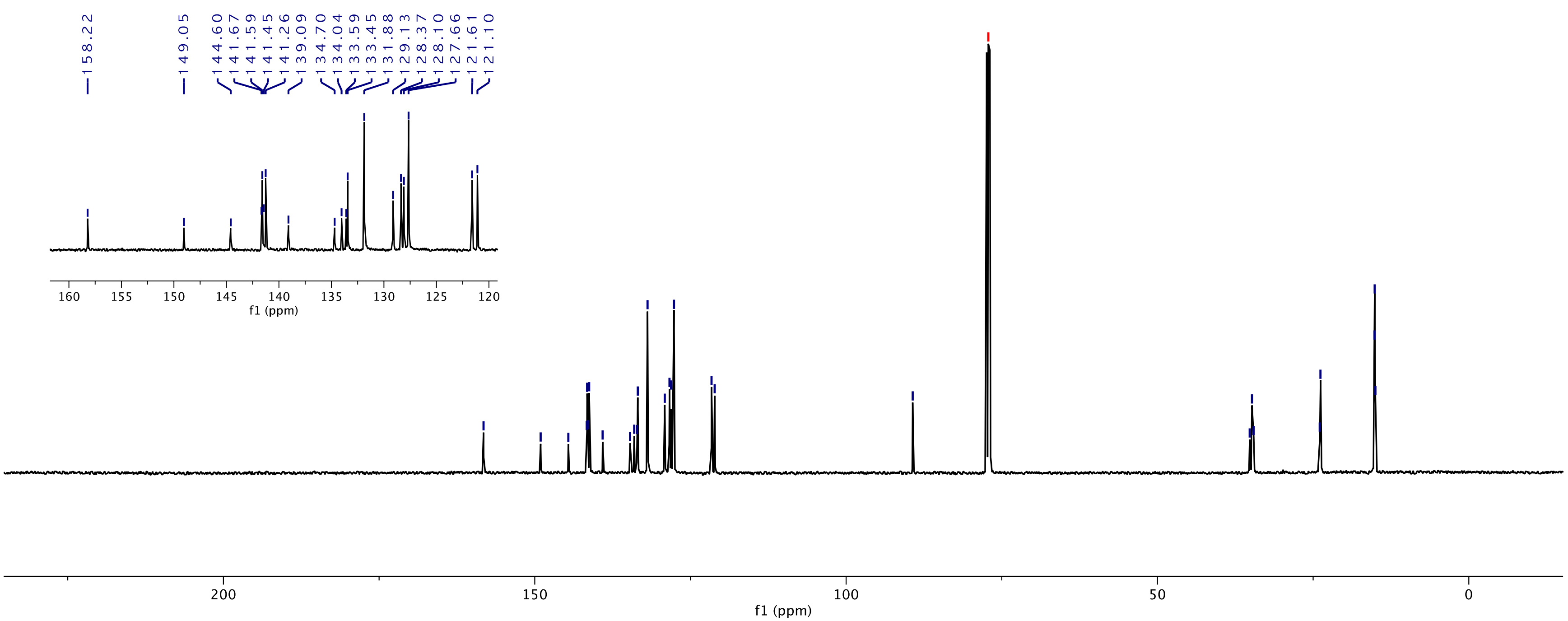

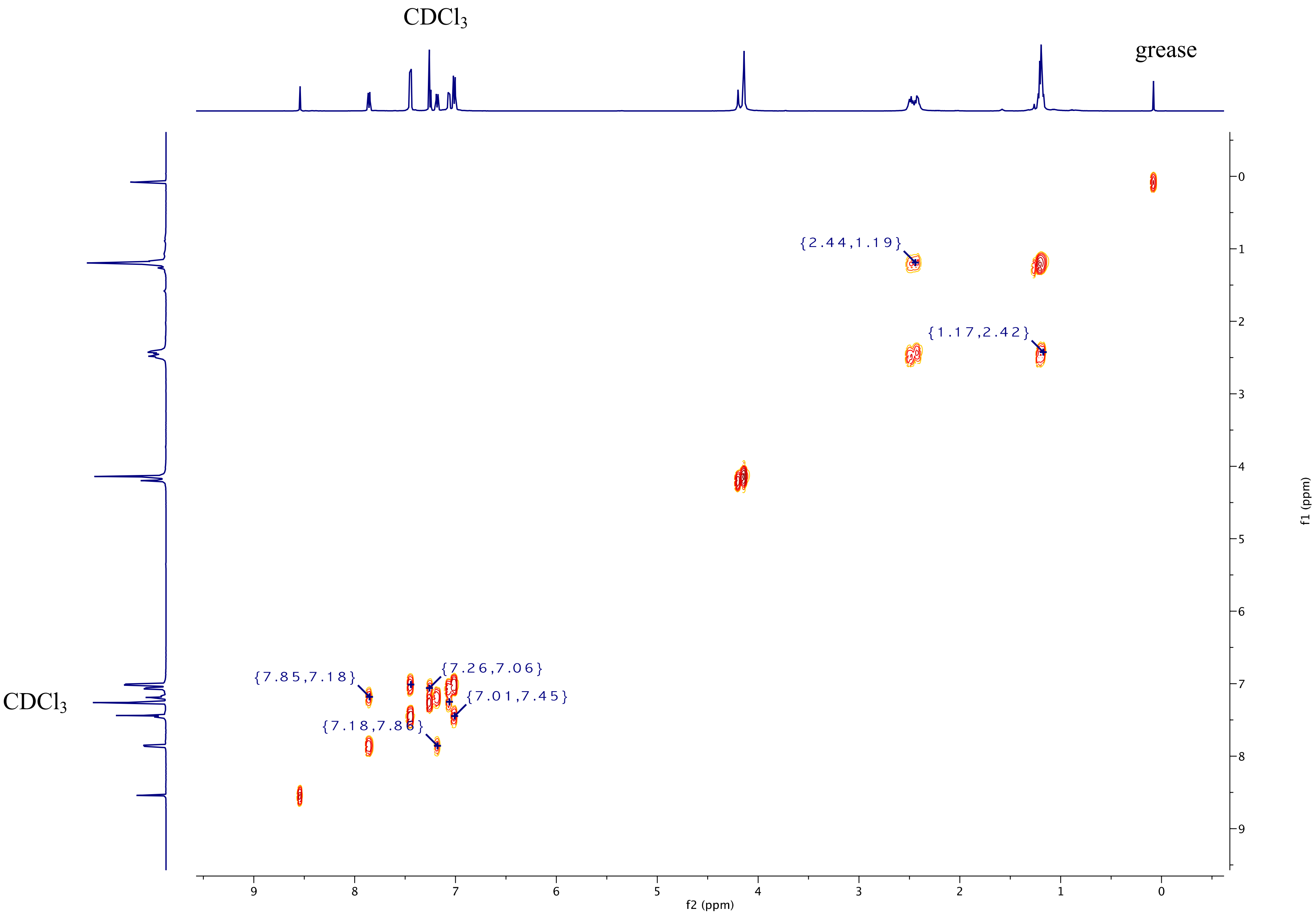

^{1}H-^{1}H COSY Spectrum of Cage **4.**

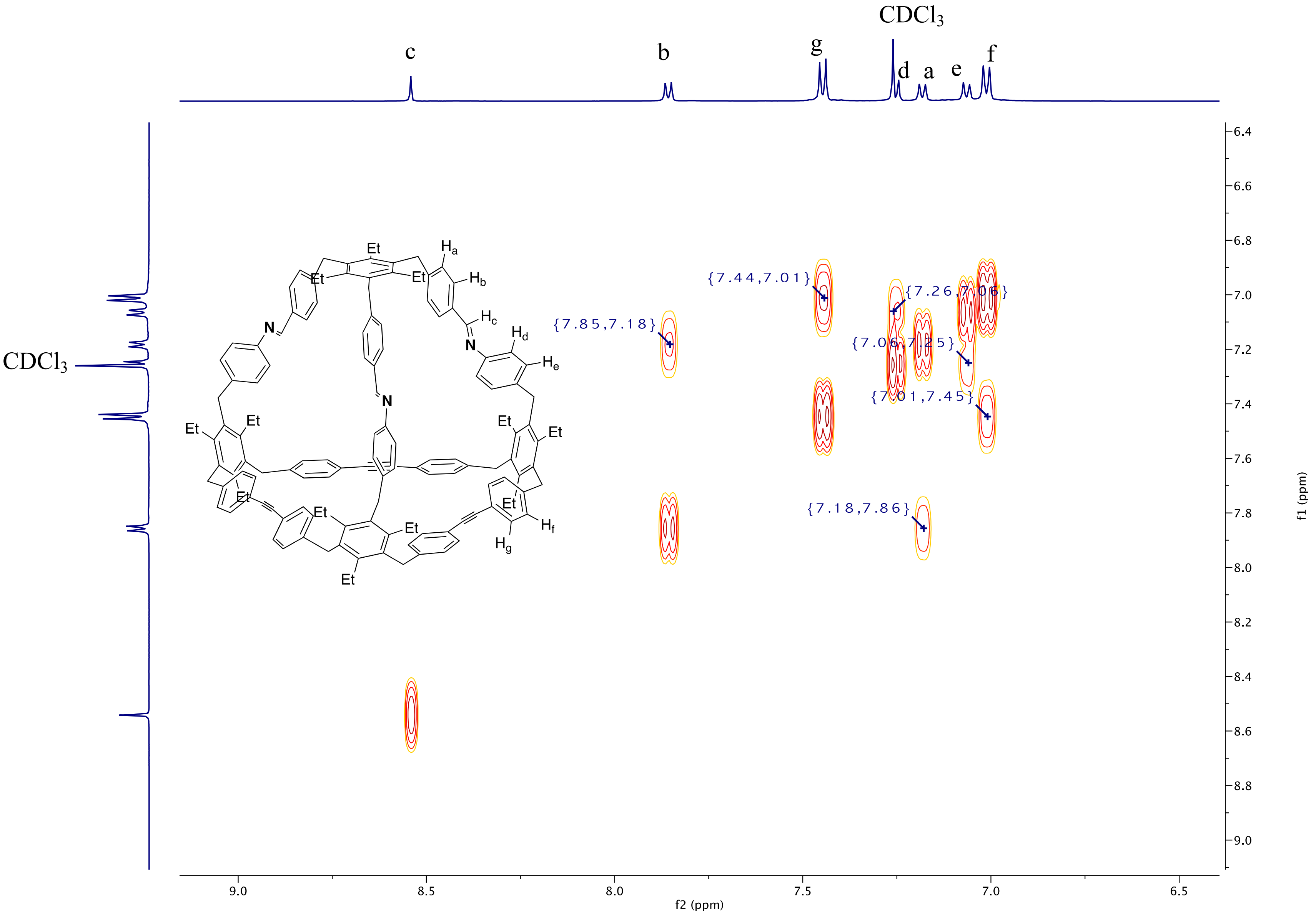

^{1}H-^{1}H COSY Spectrum of Cage **4** (aromatic region).

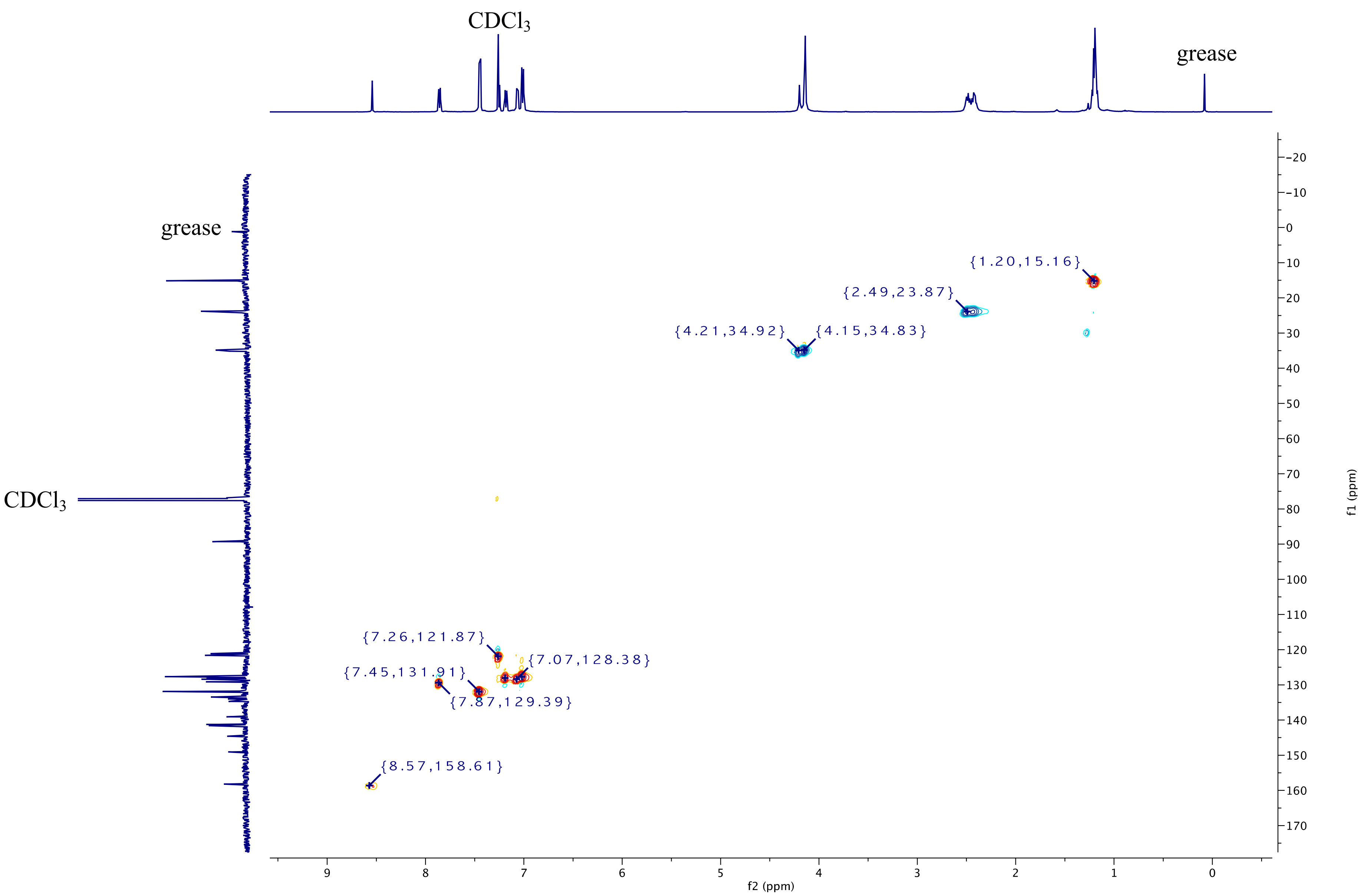

^{1}H-^{13}C HSQC Spectrum of cage **4** (500 MHz, $CDCl_3$).

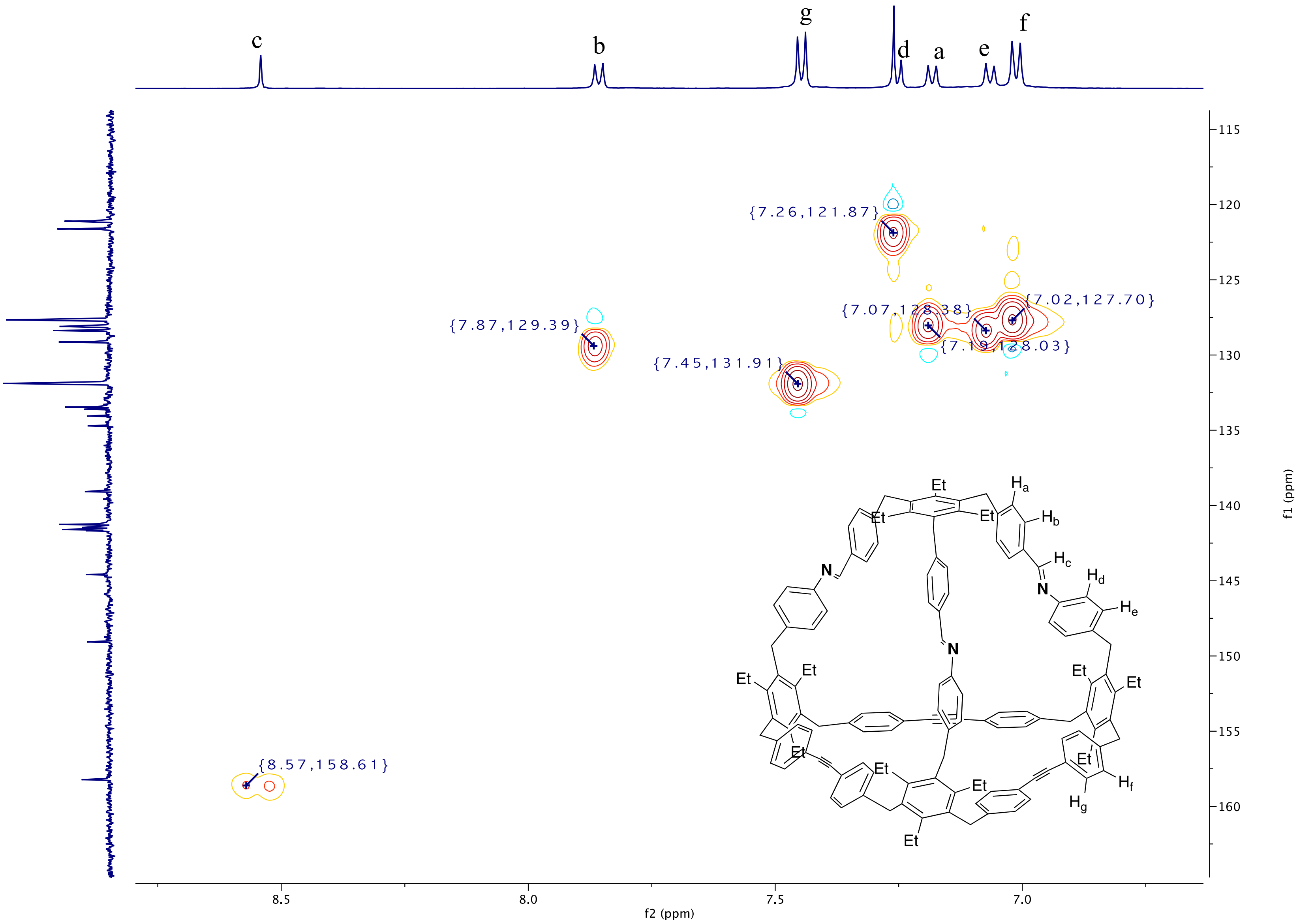

^{1}H-^{13}C HSQC Spectrum of cage **4** (500 MHz, $CDCl_3$).

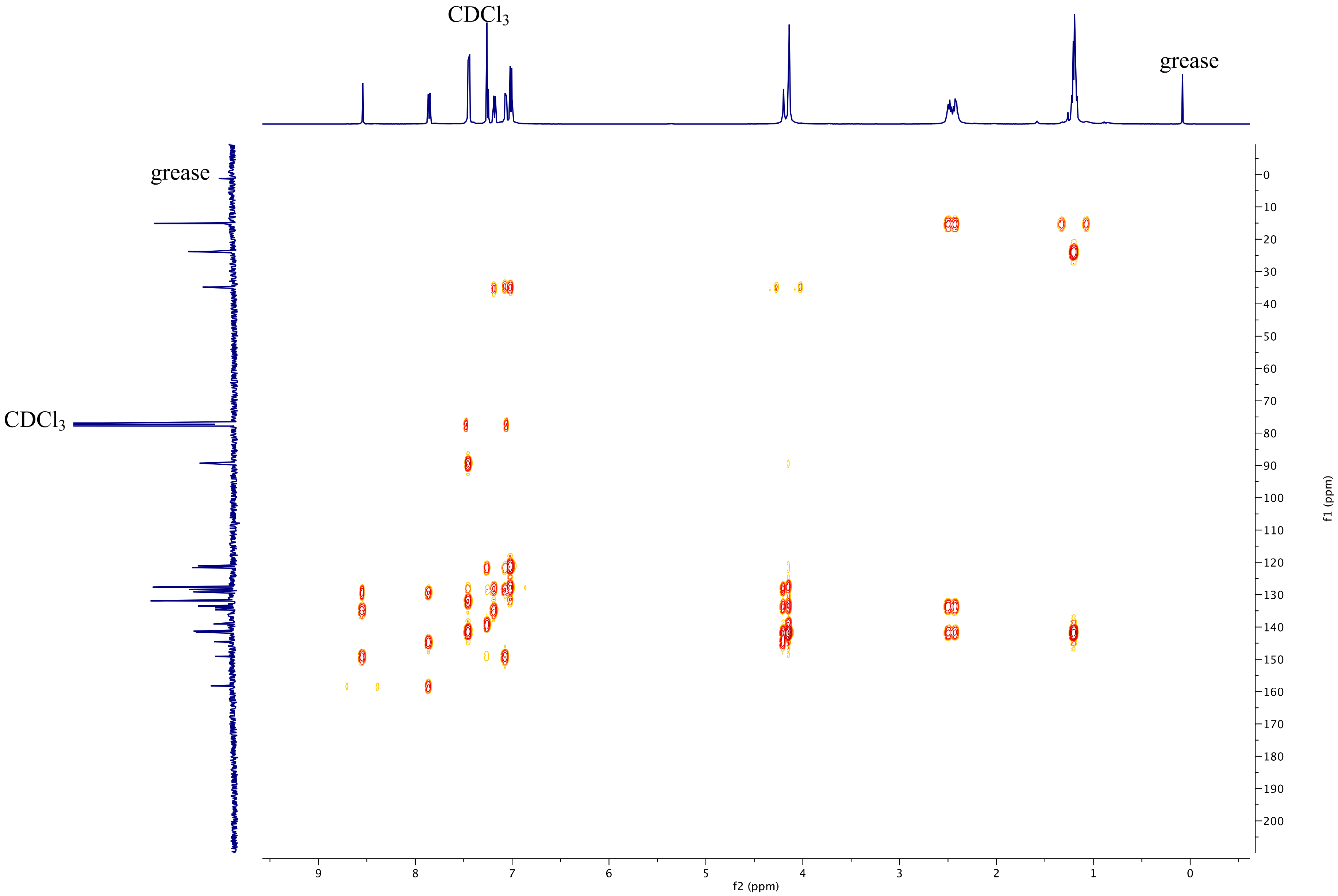

^{1}H-^{13}C HMBC spectrum of cage **4** (500 MHz, $CDCl_3$).

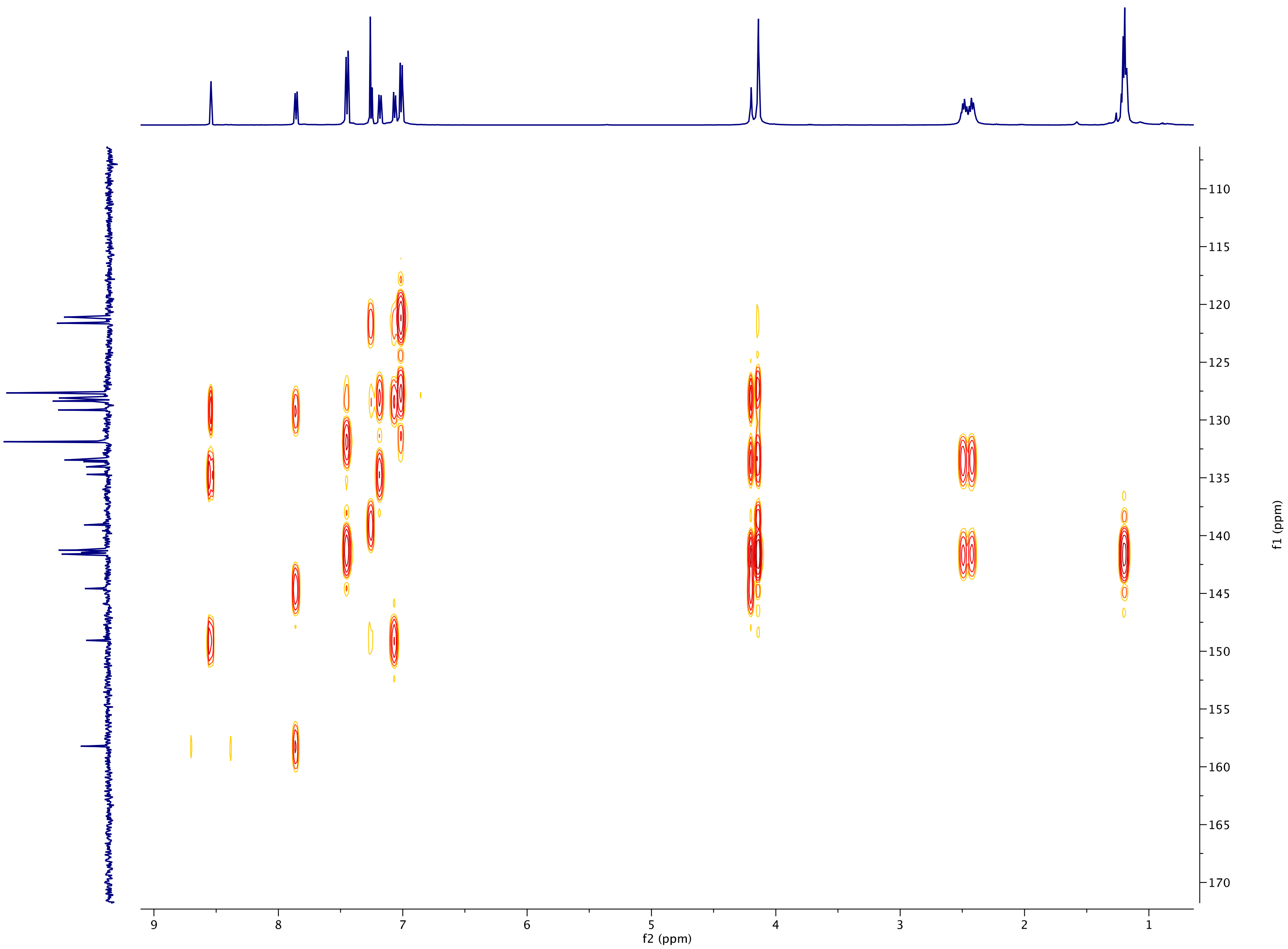

^{1}H-^{13}C HMBC spectrum of cage **4** (500 MHz, $CDCl_3$).

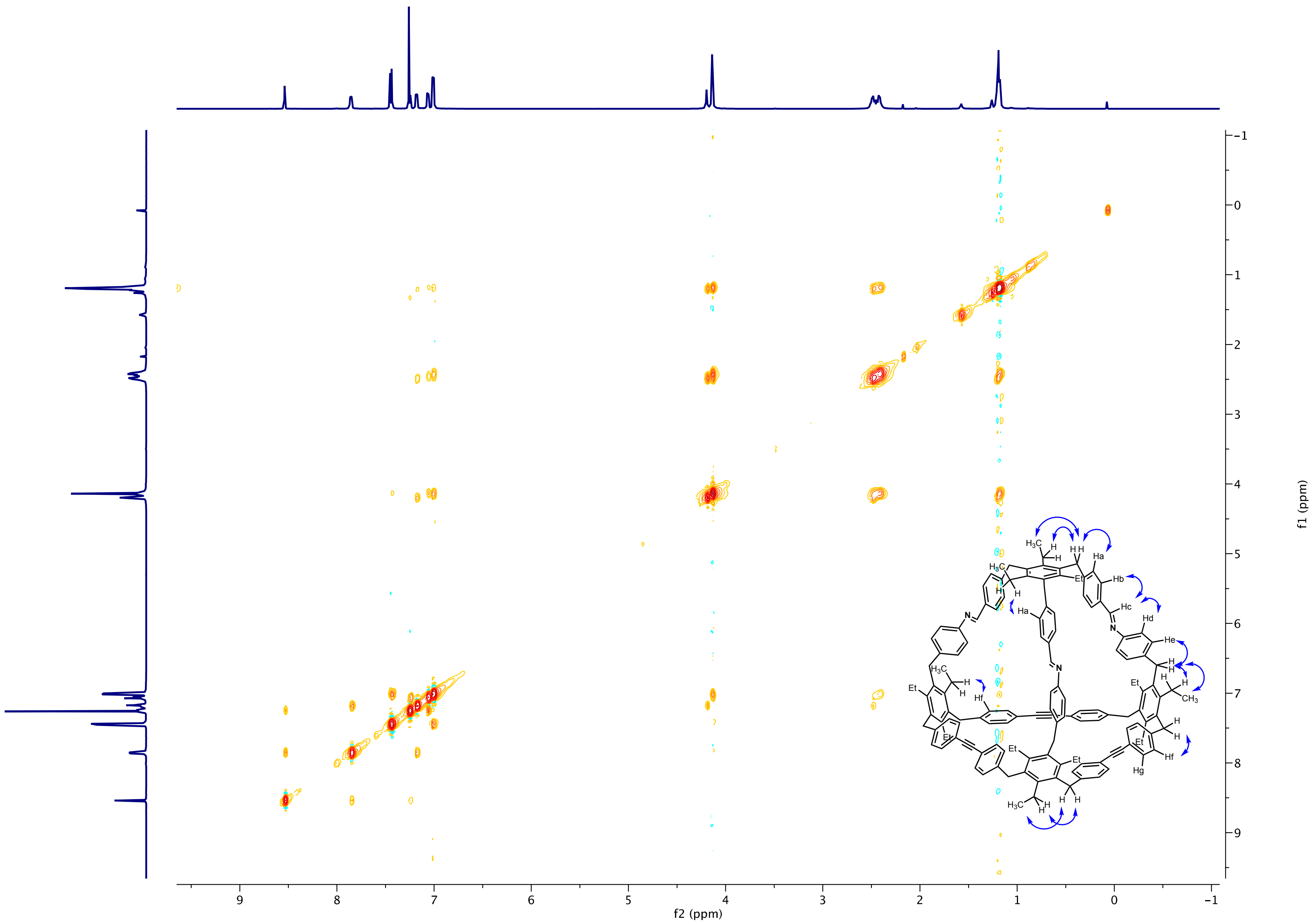

NOESY spectrum of cage **4** ($CDCl_3$, 500 MHz).

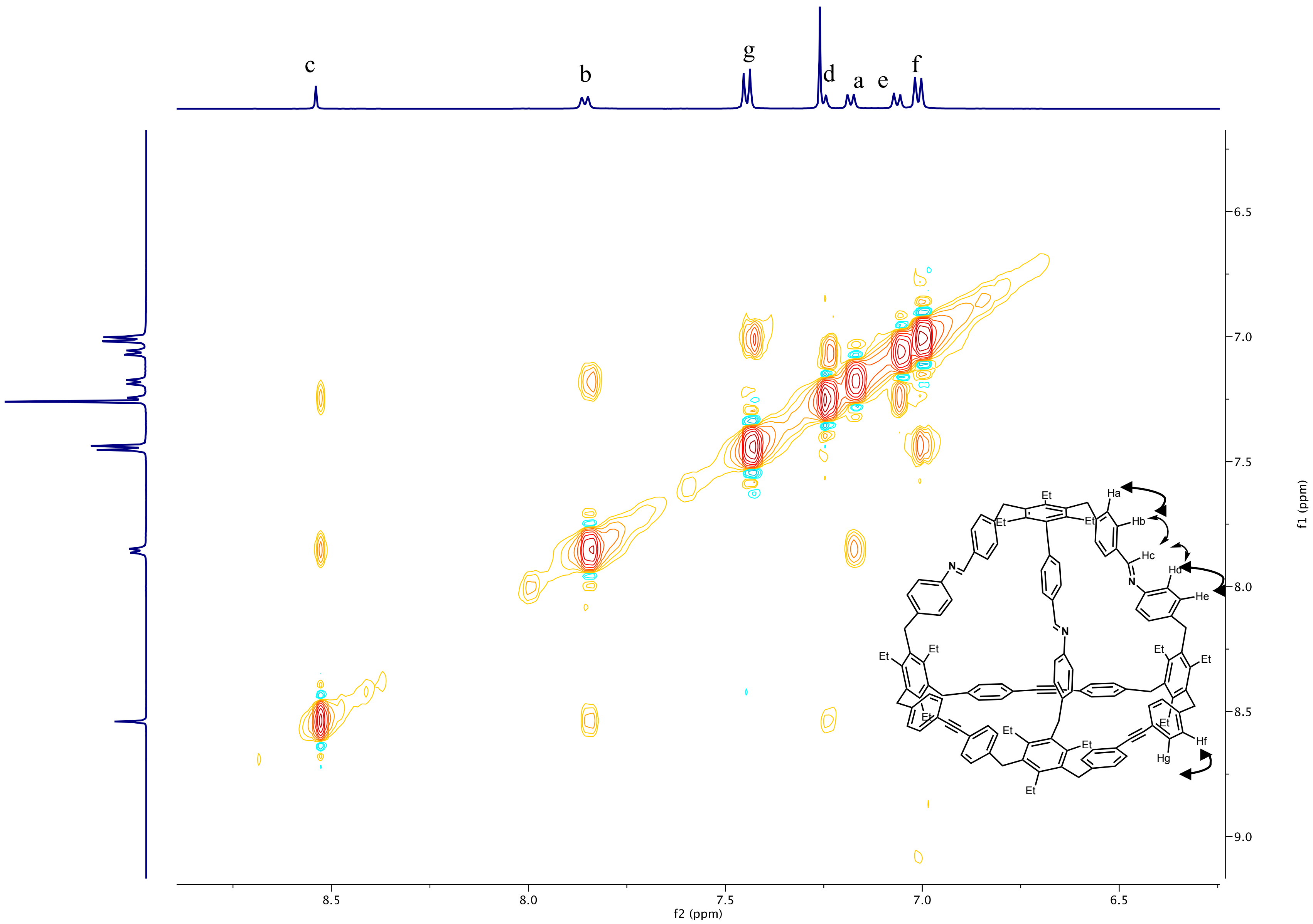

NOESY spectrum of cage **4**

S.5 References

(1) Zhang, W.; Lu, Y.; Moore, J. S. Preparation Of a Trisamidomolybdenum(VI) Propylidyne Complex. *Org. Synth.* **2007**, *84*, 163.

(2) Du, Y.; Yang, H.; Zhu, C.; Ortiz, M.; Okochi, K. D.; Shoemaker, R.; Jin, Y.; Zhang, W. Highly Active Multidentate Ligand-Based Alkyne Metathesis Catalysts. *Chem. Eur. J.* **2016**, *22*, 7959–7963.

(3) Heppekausen, J.; Stade, R.; Goddard, R.; Fürstner, A. Practical New Silyloxy-Based Alkyne Metathesis Catalysts with Optimized Activity and Selectivity Profiles. *J. Am. Chem. Soc.* **2010**, *132*, 11045–11057.

(4) Giuseppone, N.; Schmitt, J. L.; Schwartz, E.; Lehn, J. M. Scandium(III) Catalysis of Transimination Reactions. Independent and Constitutionally Coupled Reversible Processes. *J. Am. Chem. Soc.* **2005**, *127*, 5528–5539.

(5) Unusual Analyte-Matrix Adduct Ions and Mechanism of Their Formation in MALDI TOF MS of Benzene-1,3,5-Tricarboxamide and Urea Compounds. *J. Am. Soc. Mass Spectrom.* **2013**, *24*, 1405-1412.
